# Preparation of new alkyne-modified ansamitocins by mutasynthesis

**DOI:** 10.3762/bjoc.10.49

**Published:** 2014-03-03

**Authors:** Kirsten Harmrolfs, Lena Mancuso, Binia Drung, Florenz Sasse, Andreas Kirschning

**Affiliations:** 1 Institute of Organic Chemistry and Center of Biomolecular Drug Research (BMWZ), Leibniz University Hannover, Schneiderberg 1b, 30167 Hannover, Germany; 2 Department of Chemical Biology, Helmholtz Center for Infectious Research (HZI), Inhoffenstraße 7, D-38124 Braunschweig, Germany

**Keywords:** ansamitocins, antibiotics, antitumor agents, click chemistry, mutasynthesis, natural products

## Abstract

The preparation of alkyne-modified ansamitocins by mutasynthetic supplementation of *Actinosynnema pretiosum* mutants with alkyne-substituted aminobenzoic acids is described. This modification paved the way to introduce a thiol linker by Huisgen-type cycloaddition which can principally be utilized to create tumor targeting conjugates. In bioactivity tests, only those new ansamitocin derivatives showed strong antiproliferative activity that bear an ester side chain at C-3.

## Introduction

Although natural products and analogues cover a large portion of the drug market particularly in the treatment of cancer as well as bacterial and viral infections their role in pharmaceutical research has decreased over the past two decades. In part, this development is due to their often limited accessibility as well as their structural complexity. This situation hampers their use as lead structures for which access to small compound libraries is essential in order to perform structure–activity relationship studies. Besides semisynthetic and total synthesis approaches the combination of chemical synthesis with biotechnological strategies has seen increased interest lately [1–3]. The concepts either rely on a concise understanding of the biosynthesis of natural products or simply individual enzymes for in vitro applications [4]. In this context, producer strains that are genetically engineered in the biosynthesis of important and complex natural products have shown to be powerful synthetic tools, e.g., knock out mutants are key players in mutational biosynthesis, or in short mutasynthesis. This technique relies on the cellular uptake of modified biosynthetic intermediates. Processing of these intermediates, sometimes called mutasynthons, can provide complex secondary metabolites specifically modified as planned by choice of the synthetic modification incorporated into the mutasynthon [5–7].

In earlier work, we demonstrated that the ansamitocins (maytansinoids) **3**–**5** are an ideal showcase for creating small libraries by mutasynthesis [8–10]. These secondary metabolites exert strong antiproliferative activity towards different leukemia cell lines as well as human solid tumors. Inhibitory concentrations were as low as 10^−3^ to 10^−7^ µg/mL [11] which resulted from binding to β-tubulin monomers [12]. The ansamitocins are produced by *Actinosynnema pretiosum.* For our mutasynthetic studies we utilized an AHBA blocked mutant of *A. pretiosum* [13–21]. 3-Amino-5-hydroxybenzoic acid (**1**, AHBA) is the starter building block of the PKS type I that is responsible for the biosynthesis of the ansamitocin backbone [22]. This PKS is a well studied megaenzyme complex and after the action of tailoring enzymes succeeding the PKS machinery ansamitocins **3**–**5** are formed [23–27].

**Scheme 1:**
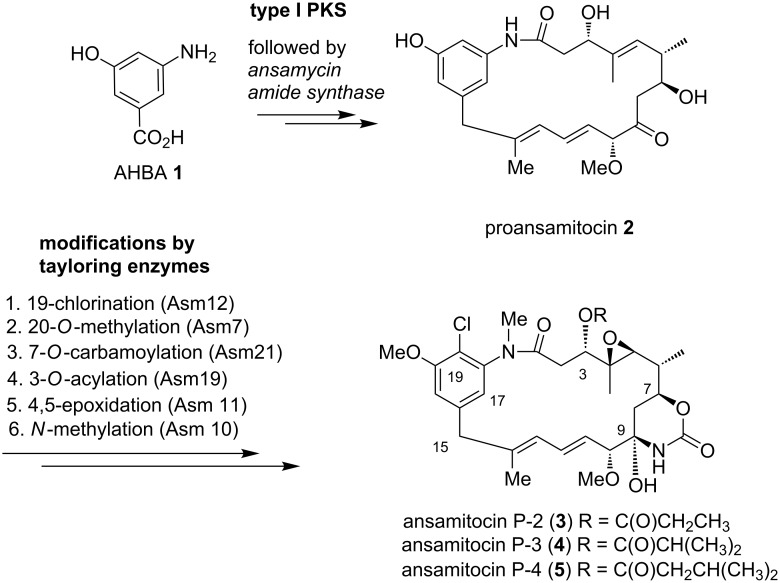
Short representation of ansamitocin biosynthesis.

In the present case, **1** is loaded on the starter module of the polyketide synthase (Scheme 1). The last PKS module releases and cyclizes *seco*-proansamitocin, likely by an ansamycin amide synthase (gene *asm9*) [23–27], that generates the 19-membered macrocyclic lactam proansamitocin (**2**). From here a set of tailoring enzymes transform proansamitocin into the bioactive final metabolites **3**–**5** following a predetermined logic that is only flexible in part (Scheme 1).

**Scheme 2:**
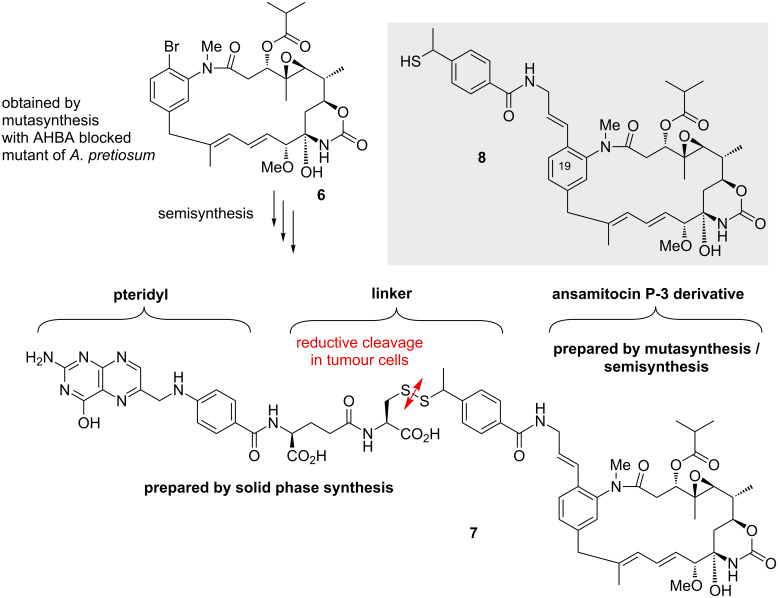
Structures of bromo-ansamitocin derivative **6**, folate-ansamitocin P-3 conjugate **7** and thiol **8**.

We extended these studies by combining mutasynthesis and semisynthesis and thus accessed four new tumor specific folic acid/ansamitocin conjugates [28] (Scheme 2). Bromo-ansamitocin **6** was obtained by mutasynthesis and was synthetically modified to the complex folic acid/drug conjugate **7**. The vitamin folic acid has become a promising ligand for selectively targeting the folate receptor (FR) in cancer tissues where the FR is known to be overexpressed [29–30]. Folic acid has a high affinity for the FR (*K*_d_ = 10^−10^ M), even when conjugated to a cytotoxin such as maytansin. An important feature of these conjugates is the linker concept that connects the drug to the tumor-specific ligand. The linker is commonly designed in a way that a release mechanism of the cytotoxin is part of the molecular architecture of the conjugate [31].

Disulfide linkers have shown to be well suited when utilizing the reducing power between extra- and intracellular milieus which results in cleavage and liberation of the drug. Mitomycin conjugates [32] were one of the earliest examples of folate disulfide–drug conjugates and after the conjugate is internalized by endocytosis, it was demonstrated that the endosomes exert reductive cleavage.

For conjugate **7** we found that disulfide cleavage provided a thiol derivative of ansamitocin P-3 (**4**, AP-3) **8** with still strong antiproliferative activity (IC_50_ < 10 nM) for different cancer cell lines. Importantly, the intact conjugate showed strong antiproliferative activity for a FR+ cancer cell line but was inactive against a FR− cell line. Indeed, despite the substantial size of the substituent remaining at C19 after reductive cleavage, ansamitocin derivative **8** still showed sufficient antiproliferative activity. This is in line with our observation that structural changes at the aromatic ring do not affect the biological properties of the ansamitocins to a great extent [13–21].

In order to broaden the opportunities of this approach for the ansamitocins, we now describe alternative accesses towards disulfide linked conjugates that are based on thiol-functionalized AP-3 derivatives. These are planned to be obtained by a combined muta- and semisynthetic strategy using different aminobenzoic acids as mutasynthons.

## Results and Discussion

### Mutasynthetic experiments

**Scheme 3:**
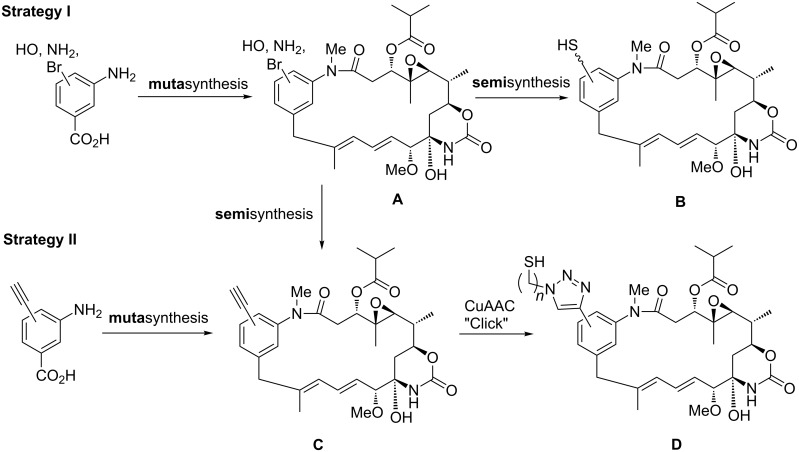
Strategies for introducing linker-based thiol groups to the aromatic moiety of ansamitocin P-3 for accessing tumor targeting conjugates (CuAAC; Cu-mediated azide–alkyne cycloaddition).

As potential groups for introducing linker systems bound to the aromatic moiety that contain terminal thiol groups (**B** and **D**, Scheme 3), we envisaged several activated functional groups (Hal, OH, NH_2_). Activation was planned to be achieved by benzylic positioning as well as by choosing aryl bromides **A** (Scheme 3) that can be modified to the corresponding aryl alkynes **C** by cross coupling chemistry. Previously, we successfully utilized this combination of mutasynthesis and semisynthesis for 19-brominated ansamitocin derivatives [13]. Alternatively, also alkyne-, vinyl- and allyl-substituted aminobenzoic acids can serve as mutasynthons so that the corresponding alkynyl- or respectively alkenyl-substituted ansamitocin derivatives can be accessed directly after fermentation. Alkynes can be further used for Huisgen-type cycloadditions better known as “copper mediated Click-chemistry” while alkenes, especially vinyl- and allylbenzoic systems, are predestined for being utilized in cross metathesis reactions.

**Figure 1:**
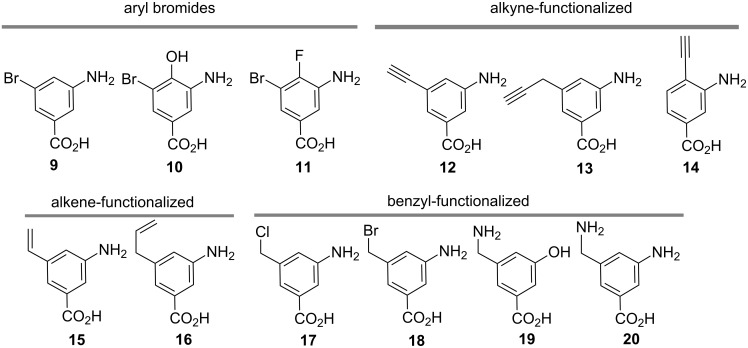
*m-*Aminobenzoic acid derivatives **9**–**20** tested as mutasynthons.

Therefore a series of aminobenzoic acids **9**–**20** (Figure 1) were prepared (see Supporting Information File 1). We expected them to serve our purposes, when fully processed after being fed to the mutant strain of *A. pretiosum* blocked in the biosynthesis of the PKS starter unit AHBA (**1**). We found that several of these aminobenzoic acids, namely **10**, **14** and **16**–**20** were either not loaded onto the PKS or not processed by the polyketide synthase in *A. pretiosum* and thus no formation of new ansamitocin derivatives was encountered in these cases.

**Scheme 4:**
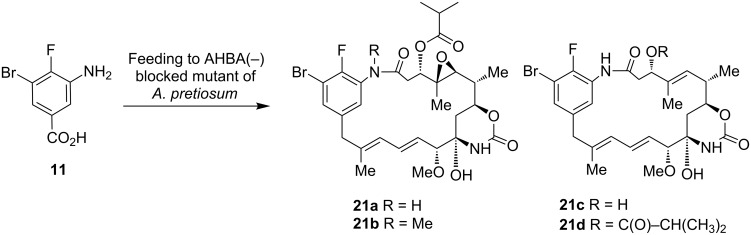
Mutasynthetic transformation of aminobenzoic acid **11** with AHBA(−)-mutant of *A. pretiosum*; putative structures of ansamitocin-derivatives **21a**–**d** as judged from HRMS analysis.

In contrast, benzoic acid **11** provided Br-F-ansamitocin derivatives **21a**–**d** after being fed to a growing culture of the mutant strain as judged by HRMS (Scheme 4). The retention times in LC and MS experiments clearly showed common patterns for ansamitocins. Additionally, the isotopic pattern provided evidence for the incorporation of the bromo functionality (see Supporting Information File 1). However, yields for each of the four ansamitocins were too small for practical scale-up.

**Scheme 5:**
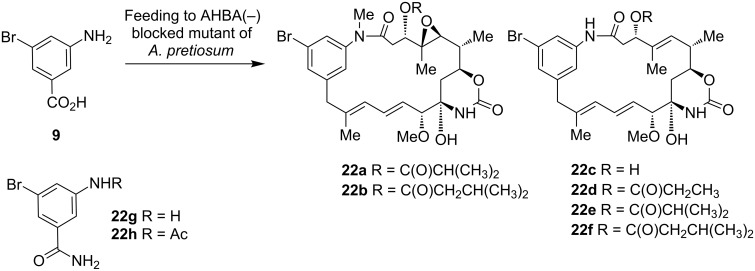
Mutasynthetic transformation of aminobenzoic acid **9** with AHBA(−)-mutant of *A. pretiosum* to bromo-ansamitocins **22a**–**f** and detoxification products **22g** and **22h**.

To evaluate the sterical and electronical properties of mutasynthon **11** with respect to bioprocessing, 3-amino-5-bromobenzoic acid (**9**) was next employed in feeding experiments. It was well processed to a series of new ansamitocin derivatives **22a**–**f** (Scheme 5).

**Scheme 6:**
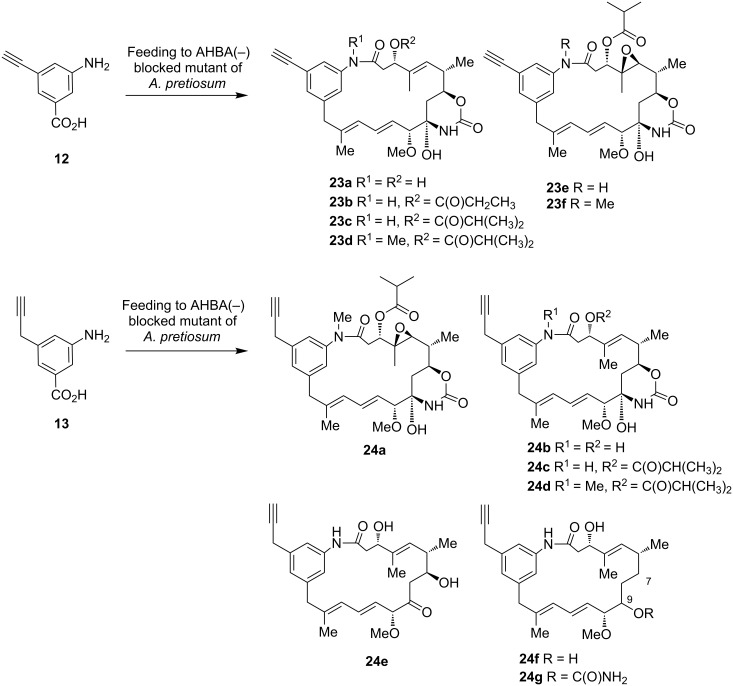
Mutasynthetic transformation of aminobenzoic acids **12** and **13** with AHBA(−)-mutant of *A. pretiosum.*

Again, all derivatives showed common fragmentation patterns and isotopic composition in the MS. Bromo-ansamitocin derivatives **22a**, **22c** and **22e** were isolated after scale up to a 1000 mL culture broth. Structures, expected from HRMS analysis, were corroborated by NMR spectroscopy. Yields were determined to be in the low mg/L range or 0.01% yield based on compound **9** (see Supporting Information File 1 for further details). In addition to the formation of the bromo-ansamitocin derivatives, also the detoxification products **22g** and **22h** were isolated in 0.1% yield. The bromo-ansamitocins can be regarded to be an ideal starting material for transition metal-catalyzed coupling reactions. Due to the low product yield obtained with mutasynthon **9**, for which the steric demand of the bromo substituent can likely be made responsible, we switched the strategy towards the direct introduction of an alkyne moiety by a mutasynthetic approach. Feeding of alkynyl(amino)benzoic acid **12** to cultures of the AHBA(−)-mutant of *A. pretiosum* yielded six new alkynyl-modified ansamitocin derivatives **23a**–**f** in yields in the lower mg/L range (Scheme 6). Among them, the major compound **23f** was obtained in 0.34% yield based on compound **12**, furnishing enough mutasynthetic material (15 mg) for further chemical transformations (see below).

Likewise, also feeding of propargyl-substituted aminobenzoic acid **13** furnished new propargyl-modified ansamitocins **24a–g**. Besides the expected AP-3 derivative **24a**, also the formation of the corresponding *N*-desmethyl ansamitocins **24b** and **24c** and/or desepoxy-derivatives **24b–d** were encountered. Finally, also the propargyl-modified proansamitocin **24e** and two truncated derivatives **24f** and **24g** were obtained. We had observed this unprecendeted defunctionalization at C7 in proansamitocin and the possible biotransformation before for the mutasynthon 5-chloro-3-aminobenzoic acid [30]. Again, reduction of the keto group at C9 occurred; but the relative configuration of C9 also remains unknown in the present example. All derivatives, which could be isolated in preparative scale (**24b**–**d**, **24f** and **24g**) were formed in 0.1% scale.

In essence, the loading module of the ansamitocin PKS shows excellent flexibility towards aminobenzoic acids that bear the slim alkynyl or propargyl substituent at C5. After upscaling of the fermentation alkynyl-ansamitocins **23c**, **23f**, **24b–d** and **24f** and **24g** were isolated and the structures were corroborated by analysis of NMR and MS spectra. Derivatives **23a**, **23b**, **23d**, **23e** and **24a**, **24e** were detected only by HRMS–MS analysis of the crude extract.

**Scheme 7:**
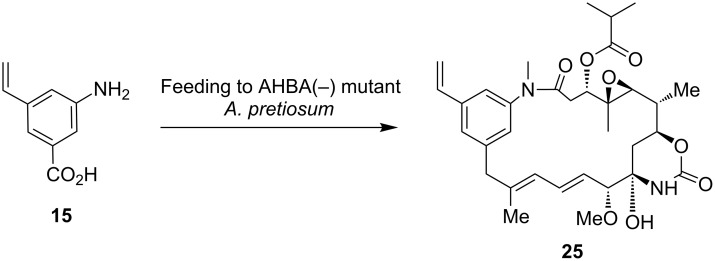
Mutasynthetic transformation of vinyl(amino)benzoic acid **15** with AHBA(−)-mutant of *A. pretiosum*.

Mutasynthetic experiments with vinyl(amino)benzoic acid **15** supposedly provided ansamitocin derivative **25** as judged by HRMS–MS analysis of the crude extract (Scheme 7). However, yields were too low for practical upscaling.

**Scheme 8:**
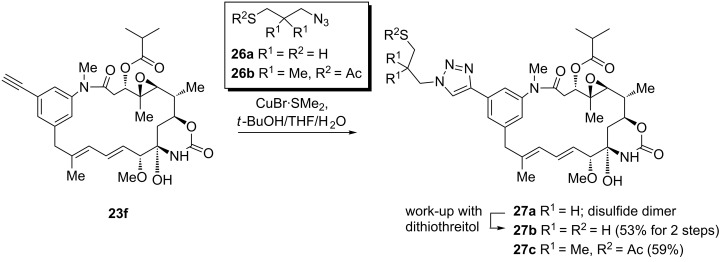
Preparation of thiofunctionalized ansamitocin derivatives **27** by Huisgen-type copper-mediated cycloaddition.

As mutasynthetic experiments with alkynyl- and propargyl(amino)benzoic acids gave best results, we decided to test the Huisgen-type cycloaddition with alkynyl derivative **23f** bearing the biologically relevant ester side chain, the oxirane moiety and the carbamoyl group for introducing a thiol moiety that would be suited for further conjugation. Two linker elements **26a** and **26b** were prepared (see Supporting Information File 1) and coupled with ansamitocin derivative **23f** to yield the disulfide dimer **27a** and the thioacetate **27c** (Scheme 8). The former could be transformed into the thiol monomer **27b** by dithiothreitol reduction.

### Biological testing

New ansamitocin derivatives **22a**, **22c**, **22e**, **23c**, **23f**, **24b–d**, **24f**, **24g**, **27b** and **27c** were subjected to in vitro biological testing with different human cell lines and one murine cell line derived from tumors or from connective tissue, respectively. The results are listed in Table 1 and are provided as values for the half-maximal inhibitory concentration of the respective ansamitocin derivatives. The most active derivatives were **23f** and **27c**. Expectedly, **24b** and **24f** are inactive, as they lack the ester side chain at C3, the key pharmacophore of the maytansinoids. All other derivatives show strong to moderate antiproliferative activity, irrespective whether the lack of the *N*-methyl group, and or the oxirane groups or not. This is in line with the view obtained from structure–activity relationship studies that these tailoring modifications only modulate the biological activity of the maytansinoids. In the present study, the human cancer cell lines were more sensitive than the mouse fibroblasts. Some derivatives also seem to exert a specificity towards certain cell lines, e.g. A-431 showed particular sensitivity for **24g**.

**Table 1:** Antiproliferative activity IC_50_ [ng/mL] of **22a**, **22c**, **22e**, **23c**, **23f**, **24b–d**, **24f**, **24g**, **27b** and **27c** in comparison to AP-3 (**4**). Values shown are means of two determinations in parallel; human cell lines: KB-3-1 (cervix carcinoma), A-431 (epidermoid carcinoma), SK-OV-3 (ovary adenocarcinoma), PC-3 (prostate adenocarcinoma), L-929 (connective tissue of a mouse).

cell line	KB-3-1	A-431	SK-OV-3	PC-3	L-929
compound					

AP-3 **4**	0.11	0.050	0.030	0.035	0.1
**22a**	2.1	0.31	0.34	0.26	4.1
**22c**	0.28	0.65	3.1	4.0	78
**22e**	7.5	5.1	1.8	0.80	8.7
**23c**	2.2	0.77	0.46	0.28	1.9
**23f**	0.38	0.074	0.099	0.14	0.81
**24b**	>10000	>10000	>10000	>10000	>10000
**24c**	7.8	5.5	9.0	18	220
**24d**	6.0	70	8.0	95	580
**24f**	1800	2200	2500	3500	>10000
**24g**	50	0.85	70	3.8	100
**27b**	2200	>10000	2800	>10000	>10000
**27c**	0.09	0.25	0.09	0.28	0.33

The high antiproliferative activity of the “click” product **27c** is remarkable as it demonstrates that substantial structural changes at C20 of the ansamitocins are tolerated and thus alkyne substituents open up diverse opportunities to diversify that position as reported including the introduction of tumor targeting ligands. Noteworthy **27b** is inactive which we ascribe to the fact that it may have dimerized back to **27a**.

## Conclusion

The combination of mutasynthesis and semisynthesis has great potential for the production of more selective ansamitocin derivatives. In this work we pursued different options to prepare ansamitocin derivatives by mutasynthesis that are set for introducing linker systems to the aromatic moiety suited to create conjugates composed of ansamitocin and tumor-specific ligands.

## Supporting Information

The supporting information provide the synthesis of building blocks, mutasynthetic experiments as well as purification protocols after fermentations, a short description of the cell proliferation assay, analytical descriptions of new metabolites and copies of ^1^H and ^13^C NMR spectra.

File 1 Additional experimental details and NMR spectra.
