# Supplementary material for: Preparation of new alkyne-modified ansamitocins by mutasynthesis
Source: Beilstein J Org Chem. 2014 Mar 3;10:535–43. doi: 10.3762/bjoc.10.49 (PMC3943755; doi:10.3762/bjoc.10.49)
Supplement: File 1 — Additional experimental details and NMR spectra. [file Beilstein_J_Org_Chem-10-535-s001.pdf]

Supporting information

for

Preparation of new alkyne-modified ansamitocins by mutasynthesis

Kirsten Harmrolfs[1], Lena Mancuso[1], Binia Drung[1], Florenz Sasse[2] and Andreas Kirschning[*1]

Address: [1]Institute of Organic Chemistry and Center of Biomolecular Drug Research (BMWZ), Leibniz University Hannover, Schneiderberg 1b, 30167 Hannover, Germany and [2]Department of Chemical Biology, Helmholtz Center for Infectious Research (HZI), Inhoffenstraße 7, D-38124 Braunschweig, Germany

Email: Andreas Kirschning - andreas.kirschning@oci.uni-hannover.de

*Corresponding author

Additional experimental details and NMR spectra

1. **General information**
2. **Preparation and purification protocols and analytical data**
   - **2.1 Aminobenzoic acid derivatives 12, 13 and 20**
   - **2.2 Br-F-Ansamitocin derivatives 21a–d**
   - **2.3 Br-Ansamitocin derivatives 22a–f and Detoxification products 22g,h**
   - **2.4 Alkynylansamitocin derivatives 23a–f**
   - **2.5 Propargylansamitocin derivatives 24a–g**
   - **2.6 Vinylansamitocin derivatives 25a,b**
   - **2.7 Linker 26a,b**
   - **2.8 Linker bound Ansamitocin derivatives 27b,c**
3. **Cell proliferation assay**
4. **References**

1. General information

We recorded ^{1}H NMR spectra at 400 MHz with a BRUKER Avance-400 or at 500 MHz with a BRUKER DRX-500 spectrometer at 298 K. For recording ^{13}C NMR spectra at 100 MHz a BRUKER Avance-400 and at 125 MHz a BRUKER DRX-500 instrument was used. We used following abbreviations to describe multiplicities: s = singlet, d = doublet, t = triplet, q = quartet, pt = pseudo-triplet, m = multiplet, b = broad. Commonly, we report chemical shift values of ^{1}H and ^{13}C NMR spectra in ppm relative to residual solvent signal as internal standard [S1]. The multiplicities refer to the resonances in the off-resonance decoupled spectra and were elucidated using phase-sensitive HSQC experiments. For ^{13}C NMR spectra multiplicities are given using the following abbreviations: s = singlet (due to quaternary carbon), d = doublet (methine), q = quartet (methyl), t = triplet (methylene) (br = broad). For further interpretation of the NMR spectra of ansamitocin derivatives ^{1}H-^{1}H correlations (COSY) and ^{1}H-^{13}C correlations (phase-sensitive HSQC, HMBC) experiments had to be conducted in addition. A type VG Autospec (EI) spectrometer at 75 eV (MICROMASS) and a type LCT (ESI) (MICROMASS) served to conduct basic MS measurements. The latter was equipped with a lockspray dual ion source combined with a WATERS Alliance 2695 LC system. Alternatively, a type Q-TOF premier (MICROMASS) spectrometer (ESI mode) in combination with a WATERS Acquity UPLC system equipped with a WATERS Acquity UPLC BEH C18 1.7 µm (SN 01473711315545) column (solvent A: water + 0.1% {v/v} formic acid, solvent B: MeCN or MeOH {given in experimental part} + 0.1% {v/v} formic acid; flow rate = 0.4 mL/min; gradient {*t* [min]/solvent B [%]}: {0/5} {2.5/95} {6.5/95} {6.6/5} {8/5}) was used. We give the ion mass signals (*m*/*z*) as values in atomic mass units. We performed analytical thin-layer chromatography using precoated silica gel 60 F254 plates (MERCK, Darmstadt). Spots were visualized with UV light at 254 nm or alternatively by staining with ninhydrin, permanganate or 4-methoxybenzaldehyde solutions [S2]. We performed flash column chromatography on MACHERY-NAGEL silica gel (particle size = 40–63 µm). Ansamitocin derivatives were isolated by preparative high performance liquid chromatography using a MERCK HITACHI LaChrom system (pump L-7150, interface D-7000, diode array detector L-7450 {λ = 220-400 nm, preferred monitoring at λ = 248 nm}). The following columns (abbreviations referred to in the experimental part given in parentheses) were used: (C18-SP) TRENTEC Reprosil-Pur 120 C18 AQ 5 µm, 250 mm × 8 mm, with guard column, 40 mm × 8 mm; (CN-SP) TRENTEC Reprosil 100 CN 5 µm, 250 mm × 8 mm, with guard column, 40 mm × 8 mm. Operating conditions are reported in the experimental part. We measured melting points using either a SRS OptiMelt apparatus or an ELECTROTHERMAL IA 9200 instrument. Values are uncorrected. Commercially available reagents, chromatography type or dry solvents were used as received or purified by standard techniques according to the literature [S2].

Aminobenzoic acid derivatives **9**, **17** and **18** were prepared according to ref. [S3]. **10**, **11**, **15** and **16** were prepared as described previously in ref. [S4]. 3-Aminomethyl-5 hydroxybenzoic acid (**19**) was obtained according to ref. [S5].

General parameters: Cultivation of microbial strains on agar plates was conducted in a HERAEUS incubator at 28 °C, while shake flask cultivation was performed in a multilevel NEW BRUNSWICK SCIENTIFIC INNOVA 4900 gyratory multi-shaker at 180 rpm at 28 °C.

If not otherwise noted the cultivation media were prepared using distilled water and sterilized by autoclaving: YMG medium – 10 g/L malt extract (SIGMA), 4 g/L yeast extract (BACTO), 4 g/L D(+)-glucose·H_2O; YMG agar – YMG medium plus 22 g/L agar (BACTO); K-medium, basal composition (final start concentration in the main culture corresponds to 5/6 of the values given for this medium due to dilution) – 60 g/L dextrin from maize starch (FLUKA), 30 g/L D(+)-maltose·H_2O (FLUKA), 5.25 g/L cottonseed flour (PROFLO), 5 g/L $CaCO_3$, 4.5 g/L yeast extract (BACTO), 300 mg/L K_2HPO_4 (FLUKA, TraceSelect), 2 mg/L $FeSO_4$·7H_2O. K-medium, additive – autoclaved and added separately: 3 g/L L-Valine (final start concentration in the main culture, from a 3% (w/v) stock solution).

Cultivation parameters. *Actinosynnema pretiosum* HGF073[S7] was stored as spore suspension in 40% (v/v) glycerol/water at −80 °C, and used for the inoculation of YMG agar plates. Following incubation of the plates for 5 days at 28 °C, 5–8 well-sporulated colonies were transferred to a 1.5 mL tube charged with 1 mL of sterile distilled water and filled to approx. After vortexmixing, the resulting suspension was used for the inoculation of precultures in bottom-baffled 250 mL Erlenmeyer flasks charged with YMG medium (50 mL per flask, with additional steel spring). Precultures were shaken for 2 days at 28 °C before inoculation of main production cultures. Cultivations were performed in K-medium with additives (50 mL K-medium with L-valine + 4 mL preculture + 1 drop of SAG 471 anti-foam {GE BAYER SILICONES}) using 250 mL Erlenmeyer flasks (final volume: 50–60 mL per flask, with additional steel spring). Cultures were shaken for 2 days at 28 °C before addition of mutasynthons was initiated. Productivity of the strain was monitored by parallel feeding of mutasynthons of known acceptance (e.g., natural synthon: 3-amino-5-hydroxybenzoic acid hydrochloride). Mutasynthons were dissolved in DMSO/water (preferably 1:1) and sterilized by filtration. Mutasynthons were added to production cultures either portion-wise (3 portions, 24 hour interval) or preferably continuously (drop-wise; for large-scale fermentations) over the time-course of 3.5 days using autoclavable, syringe pumpdriven feeding capillaries (BRAINTREE SCIENTIFIC BS-9000-8 syringe pump with UPCHURCH SCIENTIFIC high-purity Teflon® PFA tubing {1/16” OD, 0.1” ID} and Tefzel® connectors). Shaking was continued to a total cultivation time of 7–10 days. For detection of novel products from test cultures, samples of the culture broth (200 µL) were mixed with ethanol (200 µL), centrifuged (3 min, 4 °C) and the clear supernatant subjected to UPLC-ESI-MS analysis. Failing detection of novel products, the culture broth was extracted three times with ethyl

acetate, dried over $MgSO_4$, concentrated in vacuo, filtered over silica gel with ethyl acetate and the solvent removed in vacuo.

For isolation of novel products from large-scale fermentation, the combined fermentation broth was extracted with ethyl acetate as described above, and the crude extract was subjected to a sequence of chromatographic purifications (optional silica gel chromatography, size-exclusion chromatography, reversed phase-HPLC).

2. Preparation and purification protocols and analytical data

2.1 Aminobenzoic acid derivatives 12, 13 and 20

3-Amino-5-ethynylbenzoic acid (12)

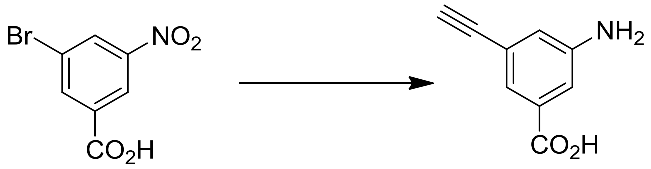

12

3-Bromo-5-nitrobenzoic acid (5.87 g, 23.6 mmol; 1 equiv), which was obtained by bromination of nitrobenzoic acid according to the protocol of **9**, was dissolved in methanol (100 mL) and a few drops of H_2SO_4 were added. The mixture was heated under refluxing conditions for 20 h before the pH was adjusted to 4 by addition of $NaOH_{aq}$. Methanol was removed in vacuo. The crude product was extracted with ethyl acetate, the organic phase was dried with $MgSO_4$, filtered and concentrated in vacuo. Methyl 3-bromo-5-nitrobenzoate was isolated (6.13g, 99%) as a light yellow solid which was directly used for the next step.

Methyl 3-bromo-5-nitrobenzoate (4.4 g, 16.9 mmol; 1 equiv) was dissolved in triethylamine (50 mL). Triphenyl phoshine (385 mg, 1.7 mmol; 0.1 equiv) was added under an argon-atmosphere before copper(I) iodide (64.8 mg, 0.34 mmol; 0.02 equiv), $Pd(dba)_2$ (195 mg, 0.34 mmol; 0.02 equiv) and trimethylsilylacetylene (4.8 mL, 33.8 mmol; 2 equiv) were added at room temperature. The mixture was stirred for 20 h at room temperature before hydrolysis with NH_4Cl_{aq} was initiated. The crude product was extracted with ethyl acetate, the organic phase was dried with $MgSO_4$, filtered and concentrated in vacuo. After rapid purification on a filter column (silica gel; petroleum ether/ethyl acetate = 10:1) methyl 3-nitro-5-ethynylbenzoate (4.6 g, 16.6 mmol; 1 equiv, 98%) was isolated and used directly for the next step.

Methyl 3-nitro-5-ethynylbenzoate (2.5 g, 12 mmol, 1 equiv) was dissolved in methanol (60 mL) and $LiOH_{aq}$ (60 mL, 1 M, 60 mmol, 5 equiv) were added. The mixture was stirred for 1 h at 60 °C before adjusting the pH to 4 with HCl_{aq} (1 M). The product was extracted with ethyl acetate, dried with $MgSO_4$, filtered and concentrated in vacuo. 3-Ethynyl-5-nitrobenzoic acid (2.2 g, 11.5 mmol, 96%) were yielded and used directly for the next step.

3-Ethynyl-5-nitrobenzoic acid (2.2 g, 11.5 mmol, 1 equiv) were dissolved in ethyl acetate (25 mL) and acetic acid (100%, few drops) and $SnCl_2*2H_2O$ (12.9 g, 57.5 mmol; 5 equiv) were added. The mixture was refluxed 2 h before poured onto ice and

the pH was adjusted to 5 with Na_2CO_{3aq}. The resulting suspension was diluted with H_2O and the product extracted with ethyl acetate. The organic phase was extracted with water and brine and finally dried with $MgSO_4$, filtered and concentrated in vacuo. 3-Amino-5-ethynylbenzoic acid (1.35 g, 8.4 mmol, 73%) was formed as a light yellow solid.

^{1}H-NMR (DMSO-d_5, 400 MHz, DMSO-d_5 = 2.50 ppm): δ_H = 7.19 (1H, s, Ar-H), 7.11 (1H, s, Ar-H), 6.84 (1H, s, Ar-H), 5.53 (2H, bs, NH_2), 4.07 (1H, s, ethynyl-H) ppm; **^{13}C-NMR** (DMSO-d_5, 100 MHz,DMSO-d_5 = 39.52 ppm): δ_C = 167.1 (s, CO), 149.2 (s, Ar-C), 131.9 (s, Ar-C), 122.2 (d, Ar-C), 120.1 (s, Ar-C), 119.6 (d, Ar-C), 115.2 (d, Ar-C), 83.5 (s, alkyne-C), 79.9 (d, alkyne-C) ppm; **HRMS** (EI) for $C_9H_7O_2N$ $[M]^+$: calculated 161.0477, found 161.0478; **mp.** = 167.8 °C.

3-Amino-5-(prop-2-yn-1-yl)benzoic acid (13)

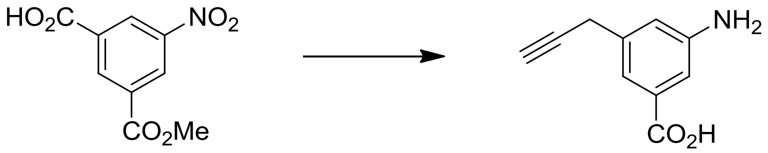

13

3-(Methoxycarbonyl)-5-nitrobenzoic acid (5.5 g, 24.4 mmol, 1 equiv) was dissolved in THF and the mixture was cooled to −10 °C. At this temperature the BH_3*SMe_2 complex, dissolved in THF (44 mL), was slowly added. The mixture was warmed up to room temperature before heating to 80 °C for 15 min. Hydrolysis with acetic $acid_{aq}$ (50%, 3 mL) terminated the reaction. The mixture was neutralized with $NaHCO_{3aq}$, THF was removed in vacuo and H_2O and ethyl acetate were added to the resulting residue. The crude product was extracted with ethyl acetate, dried with $MgSO_4$, filtered and concentrated in vacuo. The resulting crude product (6 g) was purified by column chromatography (silica gel; CH_2Cl_2/MeOH = 50:1). Methyl 3-(hydroxymethyl)-5-nitrobenzoate (4.9 g, 23.2 mmol, 95%) was isolated and the crude material was used for the next step.

Methyl 3-(hydroxymethyl)-5-nitrobenzoate (4.5 g, 21.3 mmol, 1 equiv) was dissolved in ethanol (150 mL) and palladium/charcoal (10%, 0.5 g, 0.4 mmol, 0.02 equiv) was added. The mixture was stirred for 8 h under hydrogen atmosphere before it was filtered over a pad of Celite™. The solvent was removed in vacuo. Methyl 3-amino-5-(hydroxymethyl)benzoate (3.6 g, 19.9 mmol, 86%) was isolated and directly employed in the next step.

Methyl 3-amino-5-(hydroxymethyl)benzoate (10.6 g, 58.6 mmol, 1 equiv) was dissolved in ethanol (120 mL) and Boc_2O (14.2 mL, 64.4 mmol, 1.1 equiv) was added. The mixtrure was stirred for 20 h at 30 °C before the solvent was removed in vacuo. The crude product was purified by column chromatography (silica gel, CH_2Cl_2/MeOH = 10:1). Boc-protected aniline (15.5 g, 55.1 mmol, 94%) was isolated as a colorless solid. The product was directly employed in the next step.

Boc-protected aniline (15.5 g, 55.1 mmol, 1 equiv) was dissolved in CH_2Cl_2 (220 mL), triphenylphosphine (19.5 g, 74.4 mmol, 1.4 equiv) and imidazole (5.6 g, 82.7 mmol, 1.5 equiv) was added and the mixture was cooled to 0 °C. At this temperature bromine (3.7 mL, 71.6 mmol, 1.3 equiv) was added slowly. The suspension was stirred at 0 °C for 30 min and then stirred for 2 h at room temperature. The reaction was hydrolyzed with H_2O (100 mL) and the product was extracted with CH_2Cl_2. The organic phase was extracted with $Na_2S_2O_{3aq}$ and H_2O before drying with $MgSO_4$. Filtration and concentration in vacuo yielded the crude product which was purified by column chromatography (silica gel, CH_2Cl_2/MeOH = 50:1 → 20:1). The resulting benzyl bromide (17.1 g, 49.6 mmol, 90%) was directly employed in the next step.

Trimethylsilylacetylene (4 mL, 28.4 mmol) was dissolved in THF (15 mL) under an argon atmosphere and cooled to −78 °C. At this temperature a *n*-butyllithium solution (2.5 M, hexane) was slowly added. In a second flask, indium(III) chloride (2.3 g, 10.2 mmol) was flame dried in vacuo and afterwards cooled to room temperature before suspending the salt in THF (15 mL). The suspension was cooled to −78 °C and the solution of the lithium acetylide was added dropwise to the indium suspension. The resulting reaction mixture was stirred for 30 min at −78 °C, warmed up to room temperature and stirred for additional 30 min. The benzyl bromide (5 g, 14.6 mmol, 1 equiv) was dissolved in THF (60 mL) in a third flask and Pd(dppf)Cl_2 x CH_2Cl_2 (0.22 g, 0.29 mmol; 0.02 equiv) was added. The mixture was warmed up to 65 °C and at this temperature the previously prepared indium tri(trimethylsilylacetylene) solution (10.2 mmol, 0.7 equiv) was added slowly. The mixture was stirred for 20 h at 65 °C before it was hydrolyzed by the addition of methanol. The crude product was directly adsorbed on silica gel and purified by column chromatography (silica gel; petroleum ether/ethyl acetate = 10:1 → 5:1). Methyl 3-[(*tert*-butoxycarbonyl)amino]-5-(3-(trimethylsilyl)prop-2-yn-1-yl)benzoate (4.4 g, 12.1 mmol, 83%) was isolated as a colorless oil.

^{1}H-NMR ($CDCl_3$, 400 MHz, $CHCl_3$ = 7.26 ppm): δ_H = 7.83 (1H, s, Ar-H), 7.71 (1H, s, Ar-H), 7.69 (1H, s, Ar-H), 3.90 (3H, s, CO_2Me), 3.66 (2H, s, propargylic-CH_2), 1.52 (9H, s, Boc-H), 0.20 (9H, s, TMS-H) ppm; **^{13}C-NMR** ($CDCl_3$, 100 MHz, $CDCl_3$ = 77.00 ppm): 166.7 (s, CO_2Me), 152.5 (s, Boc-CO), 138.8 (s, Ar-C), 137.8 (s, Ar-C), 131.0 (s, Ar-C), 123.7 (d, Ar-C), 122.3 (d, Ar-C), 117.7 (d, Ar-C), 103.3 (s, propargylic-2-C), 87.8 (s, propargylic-1-C), 80.9 (s, Boc), 52.2 (q, CO_2Me), 28.3 (q, Boc), 26.1 (t, propargylic-3-C), 0.0 (q, TMS) ppm; **HRMS** (EI) for $C_{19}H_{27}O_4NSi$ $[M]^+$: calculated 361.1709, found 361.1712.

Methyl 3-[(*tert*-butoxycarbonyl)-amino]-5-(3-(trimethylsilyl)prop-2-yn-1-yl)benzoate (4.4 g, 12.1 mmol, 1 equiv) was dissolved in CH_2Cl_2 (100 mL) and then cooled to −55 °C. At this temperature diisobutylaluminium hydride solution (1 M, hexane, 36.3 mL, 3 equiv) was added and the mixture stirred for 30 min at −55 °C before stirring for another 20 h at room temperature. The reaction was controlled by TLC and revealed a bit of starting material. Therefore, additional di*iso*butylaluminium hydride solution (1 M, hexane, 8 mL) was added. After 2 h the conversion was completed and the mixture was poured into an ice cold aqueous sodium-potassium tartrate solution, diluted with CH_2Cl_2 (100 mL) and vigorously stirred for 2h. After separation of the

phases the crude product was extracted with CH_2Cl_2, the organic phase was dried with $MgSO_4$, filtered and concentrated in vacuo. The crude product was purified by column chromatography (silica gel, petroleum ether/ethyl acetate = 2:1). The resulting *tert*-butyl {3-(hydroxymethyl)-5-[3-(trimethylsilyl)prop-2-yn-1-yl]phenyl}carbamate (2.9 g, 8.6 mmol, 71%) was directly employed for the next step.

tert-Butyl {3-(hydroxymethyl)-5-[3-(trimethylsilyl)prop-2-yn-1-yl]phenyl}carbamate (2.9 g, 8.6 mmol, 1 equiv) was dissolved in acetone (150 mL) and Jones reagent [S6] was added (4.3 mL). The dark red-brown solution was stirred for 4.5 h at room temperature before the starting material was consumed and the color of the solvent had turned green. The reaction was hydrolyzed by addition of isopropanol (100 mL) and stirred for 10 min before H_2O (150 mL) was added. Acetone and isopropanol were removed in vacuo before the crude product was extracted with ethyl acetate. The organic phase was dried with $MgSO_4$ and concentrated in vacuo. The resulting 3-[(*tert*-butoxycarbonyl)amino]-5-[3-(trimethylsilyl)prop-2-yn-1-yl]benzoic acid (2.7 g, 7.9 mmol, 92%) was directly employed in the next step.

3-[(*tert*-Butoxycarbonyl)amino]-5-[3-(trimethylsilyl)prop-2-yn-1-yl]benzoic acid (2.7 g, 7.9 mmol, 1 equiv) was dissolved in CH_2Cl_2 (300 mL), trifluoroacetic acid (22.3 mL, 316 mmol, 40 equiv) was added and the mixture was stirred for 4 h at room temperature. For hydrolysis the pH was adjusted to 3–4 with $NaHCO_{3aq}$. Precipitating salts were filtered off and the filtrate was extracted with ethyl acetate. The organic phase was extracted several times with H_2O before drying with $MgSO_4$ and concentration in vacuo. 3-Amino-5-[3-(trimethylsilyl)prop-2-yn-1-yl]benzoic acid (1.8 g, 7.3 mmol, 92%) was isolated and directly used for the final step.

3-Amino-5-[3-(trimethylsilyl)prop-2-yn-1-yl]benzoic acid (1.8 g, 7.3 mmol, 1 equiv) was dissolved in methanol (150 mL) and the mixture was warmed up to 50 °C. At this temperature potassium fluoride (2.1 g, 36.5 mmol, 5 equiv) was added and the mixture stirred for 6 h. After this time a second portion of KF (0.85 g, 14.6 mmol, 2 equiv) was added. After 20 h TLC analysis revealed the presence of some starting material and therefore a third portion of KF (0.85 g, 14.6 mmol, 2 equiv) was added. After 48 h the reaction mixture was hydrolyzed by addition of HCl_{aq} until a pH value of 4 was reached. Methanol was removed in vacuo and the crude product was extracted with ethyl acetate. The organic phase was dried with $MgSO_4$ and concentrated in vacuo before the oily brownish residue was dissolved with a minimum of hot HCl_{aq} (18%). Crystals were obtained by storing the saturated solution at 4 °C. 3-Amino-5-(prop-2-yn-1-yl)benzoic acid (1.3 g, 6.1 mmol, 83%) was obtained (hydrochloride salt) as colorless crystals.

^{1}H-NMR (DMSO-*d*$_5$, 400 MHz, DMSO-*d*$_5$ = 2.50 ppm): δ_H = 12.59 (1H, bs, COOH), 7.05 (1H, s, Ar-H), 7.04 (1H, s, Ar-H), 6.74 (1H, s, Ar-H), 5.35 (2H, bs, NH_2), 3.52 (2H, d, J = 2.7 Hz, Bn-CH_2), 3.05 (1H, t, J = 2.7 Hz, propargylic-H) ppm; **^{13}C-NMR** (DMSO-*d*$_5$, 100 MHz, DMSO-*d*$_5$ = 39.52 ppm): 167.8 (s, CO), 149.0 (s, Ar-C), 137.1 (s, Ar-C), 131.5 (s, Ar-C), 117.2 (d, Ar-C), 116.1 (d, Ar-C), 112.9 (d, Ar-C), 82.4 (s, propargylic-C), 73.1 (d, propargylic-C), 23.8 (t, Bn-CH_2) ppm; **HRMS (ESI)** for $C_{10}H_9NO_2$ $[M]^+$: calculated 175.0633, found 175.0632; **mp.** = 158.8 °C .

3-Amino-4-ethynylbenzoic acid (14)

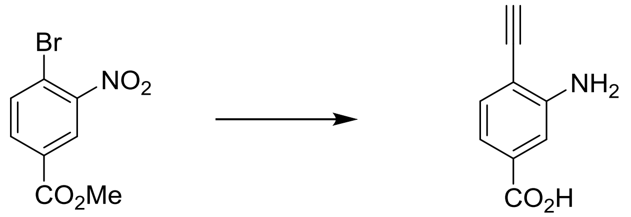

14

Methyl 4-bromo-3-nitrobenzoate (200 mg, 0.77mmol, 1 equiv) was dissolved in freshly distilled Et_3N and $Pd(PPh_3)_2Cl_2$ (7.6 mg, 0.011mmol, 0.014 equiv) and TMS-acetylene (200 µL, 1.38 mmol, 1.8 equiv) were added. The reaction mixture was stirred at 75 °C overnight, filtered through a pad of silica and washed with Et_3N. The crude product was directly adsorbed on silica gel and purified by column chromatography (silica gel; petroleum ether/ethyl acetate = 20:1). Methyl 3-nitro-4-[(trimethylsilyl)ethynyl]benzoate (156.5 mg, 0.56 mmol, 73%) was isolated as brownish crystals. The methyl ester (156.5 mg, 0.56 mmol, 1 equiv) was dissolved in ethyl acetate (20 mL) and $SnCl_2$*$2H_2O$ (631 mg, 2.80 mmol, 5 equiv) was added. The reaction mixture was stirred at 30 °C overnight, poured onto ice and the pH was adjusted to 5 with aq. Na_2CO_3. The resulting suspension was diluted with H_2O and the product was extracted with ethyl acetate. The organic phase was extracted with water and brine and finally dried with $MgSO_4$, filtered and concentrated in vacuo. Methyl 3-amino-4-[(trimethylsilyl)ethynyl]benzoate (99.3 mg, 0.40 mmol, 71%) was isolated as yellowish syrupy material.

Methyl 3-amino-4-[(trimethylsilyl)ethynyl]benzoate (30 mg, 121.3 mmol, 1 equiv) was dissolved in methanol (6 mL) and $LiOH_{aq}$ (0.68 mL, 1 M, 606.4 mmol, 5 equiv) was added. The reaction mixture was stirred at 30 °C before adjusting the pH to 4 using HCl_{aq} (1 M). The product was extracted with ethyl acetate, dried over $MgSO_4$, filtered and concentrated in vacuo to yield 3-amino-4-ethynylbenzoic acid (**14**) (19.2 g, 119.3 mmol, 98%).

^{1}H-NMR (DMSO-*d*$_5$, 400 MHz, DMSO.*d*$_5$ = 2.50 ppm): δ_H = 7.31 (1H, s, Ar-H), 7.24 (1H, s, Ar-H), 7.05 (1H, s, Ar-H), 5.60 (2H, bs, NH_2), 4.52 (1H, s, CH) ppm; **^{13}C-NMR** (DMSO-*d*$_5$, 100 MHz, DMSO-*d*$_5$ = 39.52 ppm): 167.3 (s, CO), 150.0 (s, Ar-C), 132.2 (d, Ar-C), 131.8 (s, Ar-C), 116.2 (d, Ar-C), 114.6 (d, Ar-C), 108.8 (s, Ar-C), 87.5 (t, alkyne-C), 80.4 (s, alkyne-C) ppm; **HRMS (ESI)** for $C_9H_6NO_2$ $[MH]^-$: calculated 160.0399, found 160.0409; **mp.** = 158 - 159 °C.

3-Amino-5-(aminomethyl)benzoic acid (20)

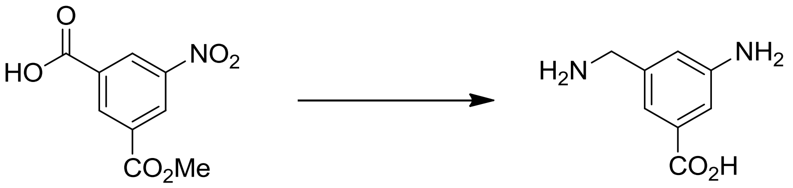

20

3-(Methoxycarbonyl)-5-nitrobenzoic acid (5.5 g, 24.4 mmol, 1 equiv) was dissolved in THF (15 mL) and cooled to −10 °C. To the cooled solution borane methyl-sulfide complex (4.3 mL) dissolved in dry THF was added dropwise. The reaction mixture was warmed up to room temperature and then stirred under refluxing conditions for 15 min. Then, the reaction mixture was cooled to room temperature, followed by addition of a mixture of acetic acid and water (1:1, 3 mL). Neutralization was achieved by the addition of saturated solution of sodium bicarbonate and the phases were separated. The aqueous phase was extracted twice with ethyl acetate and the combined organic extracts were washed with brine, dried over magnesium sulfate and evaporated. The crude product was purified by flash column chromatography (CH_2Cl_2:MeOH = 50:1) afforded methyl 3-(hydroxymethyl)-5-nitrobenzoate (4.9 g, 23.2 mmol, 95%).

The nitrobenzoate (4.5 g, 21.3 mmol, 1 equiv) was dissolved in ethanol (150 mL) and Pd/C (0.5 g, 0.4 mmol, 0.02 equiv) was added. The mixture was stirred under H_2 atmosphere for 8 h, then filtered over silica gel and concentrated in vacuo to yield methyl (3-amino)-5-hydroxymethylbenzoate (3.6 g, 19.9 mmol, 86%).

The crude product was dissolved in THF (55 mL), followed by addition of acetic anhydride (2.7 mL, 29 mmol, 1.5 equiv) and triethylamine (4.0 mL, 29 mmol, 1.5 equiv). The mixture was stirred for 6 h at room temperature. The reaction mixture was hydrolyzed by addition of a solution of saturated sodium bicarbonate which was extracted twice with ethyl acetate. The combined, organic layers were dried over $MgSO_4$ and concentrated in vacuo. Purification by recrystallization yielded the methyl 3-acetamido-5-(hydroxymethyl)benzoate (3.2 g, 14.2 mmol, 74%). The crude product (3.1 g, 13.9 mmol, 1 equiv) was suspended in CH_2Cl_2 (70 mL) and pyridine (2.47 mL, 30.6 mmol, 2.2 equiv) was added. The solution was cooled to 0 °C and thionyl chloride (2.2 mL, 30.6 mmol, 2.2 equiv) was added. The reaction mixture was stirred over night at room temperature and hydrolyzed with water. The crude product was extracted with CH_2Cl_2 twice, dried over magnesium sulfate, filtered and evaporated in vacuo. Purification by flash column chromatography (hexane:ethyl acetate = 2:1→ 1:1) afforded methyl 3-acetamido-5-(chloromethyl)benzoate (1.6 g, 6.6 mmol, 47%).

This methyl ester (1.5 g, 6.2 mmol, 1 equiv) was suspended in a mixture of acetone and water (10 mL acetone; 3 mL water) and NaN_3 (2.4 g, 37.2 mmol, 6 equiv) was added. The solution was stirred overnight under refluxing condition, then cooled to room temperature and acetone was removed in vacuo. The crude product was dissolved in dichloromethane, extracted twice with water and combined, organic

layers were dried over magnesium sulfate, filtered and concentrated in vacuo. Purification by flash column chromatography (hexane:ethyl acetate = 1:1) afforded methyl 3-acetamido-5-(azidomethyl)benzoate (1.3 g, 5.33 mmol, 86%).

Methyl 3-acetamido-5-(azidomethyl)benzoate (1.3 g, 5.33 mmol, 1 equiv) was dissolved in methanol (60 mL) and an aqueous 1 M LiOH-solution (26.7 mL, 26.7 mmol, 5 equiv) was added and stirring was continued for 4 h at 55 °C. Then, the mixture was acidified to pH 2 with 2 M HCl, the MeOH was evaporated and the resulting precipitate was extracted with ethyl acetate twice, dried over magnesium sulfate, filtered and concentrated in vacuo to yield 1.2 g of 3-acetamido-5-(azidomethyl)benzoic acid (5.33 mmol, quant.).

3-Acetamido-5-(azidomethyl)benzoic acid (1.2 g, 5.33 mmol, 1 equiv) was dissolved in water (25 mL) and 5.3 mL HCl_{conc} was added. The reaction mixture was stirred overnight under refluxing condition, then cooled to room temperature and the pH of the aqueous phase was adjusted to 5 using an aqueous solution of sodium bicarbonate. This mixture was extracted with ethyl acetate and the organic phase was dried over magnesium sulfate and concentrated in vacuo to afford 3-amino-5-(azidomethyl)benzoic acid (0.7 g, 3.5 mmol, 66%) as brown solid.

^{1}H-NMR (400 MHz, MeOD, CD_2HOD=3.310 ppm): δ_H = 7.33 (1H, bs, Ar-H), 7.28 (1H, bs, Ar-H), 6.88 (1H, bs, Ar-H), 4.29 (2H, s, CH_2) ppm. **HRMS (ESI)** for $C_8H_7N_4O_2$ [M-H]$^-$: calculated 191.0569, found 191.0569.

The aryl azide (300 mg, 1.56 mmol, 1 equiv) was dissolved in methanol (5 mL) and Pd/C (170 mg, 0.156 mmol, 0.1 equiv) and acetic acid (2 mL) were added. The mixture was stirred under an H_2 atmosphere for 72 h, then filtered over silica gel and concentrated in vacuo. Purification by flash column chromatography (ethyl acetate:methanol = 5:1 → methanol 100%) title compound (201 mg, 1.21 mmol, 78%).

^{1}H-NMR (DMSO-d_5, 400 MHz, DMSO-d_5 = 2.50 ppm): δ_H = 7.18 (1H, dd, J = 1.8, 1.6 Hz, H-Ar), 7.07 (1H, dd, J = 1.6, 1.4 Hz H-Ar), 6.60 (1H, dd, J = 1.8, 1.4 Hz H-Ar), 5.04 (2H, bs, NH_2), 3.72 (2H, s, CH_2) ppm; **^{13}C-NMR** (DMSO-d_5, 100 MHz, DMSO-d_5 = 39.52 ppm): 167.4 (s, COOH), 143.5 (s, Ar-C), 133.8 (s, Ar-C), 132.9 (s, Ar-C), 130.6 (d, Ar-C), 127.7 (d, Ar-C), 126.0 (d, Ar-C), 45.5 (t, CH_2NH_2) ppm; **HRMS (ESI)** for $C_8H_9N_2O_2$ [M H]$^-$: calc. 165.0664; found 165.0665; **mp**.= 183.3-189.2 °C.

2.2 Bromo-fluoro-substituted ansamitocin derivatives 21a–d

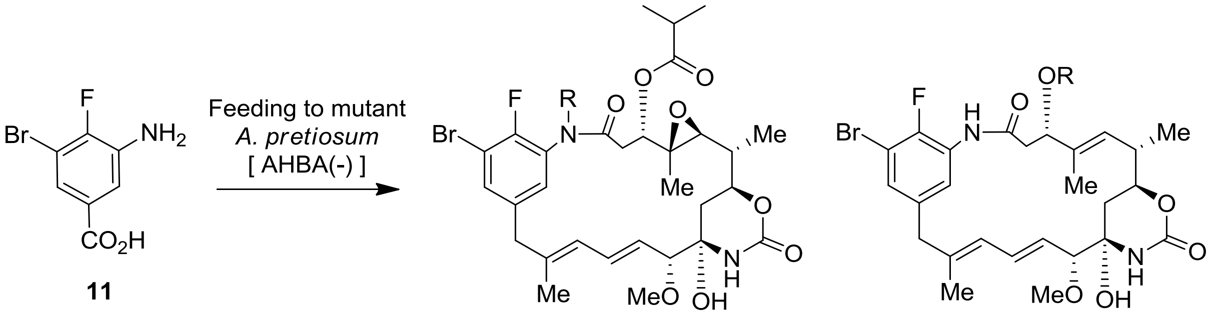

21a R= H
21b R= Me

21c R= H
21d R= C(O)-CH(CH_3)$_2$

Continuous (over 5 days) complementation of a growing culture (50 mL) of the AHBA blocked mutant of *A. pretiosum* with 3-amino-5-bromo-4-fluorobenzoic acid (**11**) (70 mg, 6 mmol/L medium) dissolved in DMSO according to the general protocol provided a crude ethyl acetate extract. This extract was prepurified by column chromatography (silica gel; ethyl acetate) and analyzed by UPLC-MS. MS-analysis indicated the formation of at least four new ansamitocin derivatives **21a-d** as judged by characteristic fragmentation patterns that have been established for the ansamitocins.

21a: HRMS(ESI) for $C_{30}H_{38}N_2O_8BrFNa$ [M + Na]$^+$: calculated 675.1693, found 675.1751.

21b: HRMS(ESI) for $C_{31}H_{40}N_2O_8BrFNa$ [M + Na]$^+$: calculated 689.1850, found 689.1843.

21c: HRMS(ESI) for $C_{26}H_{32}N_2O_6BrFNa$ [M + Na]$^+$: calculated 589.1325, found 589.1307.

21d: HRMS(ESI) for $C_{30}H_{38}N_2O_7BrFNa$ [M + Na]$^+$: calculated 659.1744, found 659.1739.

2.3 Bromo-substituted ansamitocin derivatives 22a–f

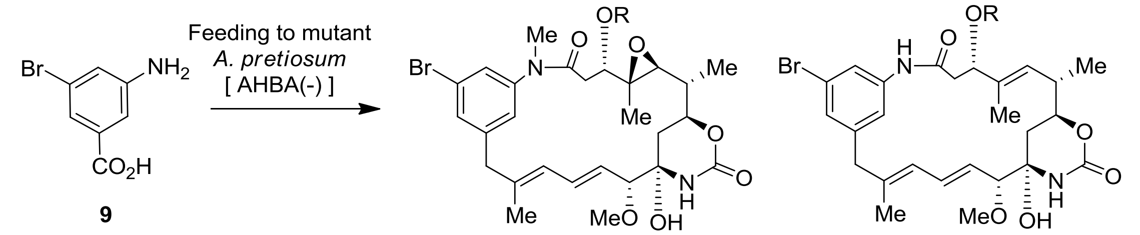

22a R= C(O)CH(CH_3)$_2$
22b R= C(O)CH_2CH(CH_3)$_2$

22c R= H
22d R= C(O)CH_2CH_3
22e R= C(O)CH(CH_3)$_2$
22f R= C(O)CH_2CH(CH_3)

Batchwise complementation of a growing culture (3 L) of the AHBA blocked mutant of *A. pretiosum* with 3-amino-5-bromobenzoic acid (**9**) (3.9 g, 6 mmol/L medium) dissolved in DMSO according to the general protocol provided a crude ethyl acetate

extract. This extract was prepurified by column chromatography (silica gel; ethyl acetate) and afterwards by HPLC (C18-SP column) using a methanol-water gradient (20:80 → 100% methanol; 90 min). The fractions were further purified by HPLC (CN-SP column, acetonitrile/water, gradient 5:95 → 100% acetonitrile; 100 min). The new ansamitocin derivatives **22a** (0.9 mg, 0.01%), **22c** (0.9 mg, 0.01%) and **22e** (0.6 mg, 0.01%) were isolated as pure compounds. MS-analysis and retention times indicated formation of three additional ansamitocin derivatives **22b**, **22d** and **22f**. These showed characteristic fragmentation patterns in the MS as established for the ansamitocins. In addition, also the detoxification products 3-amino-5-bromobenzamide **22g** and 3-acetamino-5-bromobenzamide **22h** were isolated in scales of 0.1% yield.

20-Bromo-19-deschloransamitocin P-3 (22a)

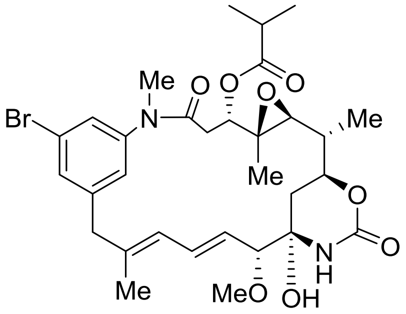

22a

^{1}H-NMR (500 MHz, $CDCl_3$, $CHCl_3$ = 7.26 ppm): δ_H = 7.40 (1H, pt, *J* = 1.6 Hz, 21-H), 7.29 (1H, pt, *J* = 1.6 Hz, 19-H), 7.14 (1H, pt, *J* = 1.6 Hz, 17-H), 6.45 (1H, dd, *J* = 15.4, 11.0 Hz, 12-H), 6.22 (1H, s, 9-NH), 6.17 (1H, d, *J* = 11.0 Hz, 13-H), 5.47 (1H, dd, *J* = 15.4, 8.9 Hz, 11-H), 4.84 (1H, dd, *J* = 12.0, 3.0 Hz, 3-H), 4.28 (1H, ddd, *J* = 12.2, 10.5, 1.9 Hz, 7-H), 3.50 (1H, d, *J* = 13.3 Hz, 15-H_a), 3.51 (1H, d, *J* = 8.9 Hz, 10-H), 3.36 (3H, s, 10-OMe), 3.22 (1H, d, *J* = 13.3 Hz, 15-H_b), 3.21 (3H, s, 1-NMe), 3.03 (1H, bs, 9-OH), 2.96 (1H, d, *J* = 9.7 Hz, 5-H), 2.68 (1H, dd, *J* = 14.1, 12.0 Hz, 2-H_a), 2.60 (1H, sep, *J* = 7.0 Hz, 2'-H), 2.22 (1H, dd, *J* = 14.1, 3.0 Hz, 2-H_b), 1.69 (3H, s, 14-Me), 1.67 – 1.62 (1H, m, 8-H_a), 1.52 - 1.44 (1H, m, 6-H), 1.29 (3H, d, *J* = 6.4 Hz, 6-Me), 1.25 (3H, d, *J* = 7.0 Hz, 3'-H_a), 1.28 – 1.22 (1H, m, 8-H_b), 1.20 (3H, d, *J* = 7.0 Hz, 3'-H_b), 0.87 (3H, s, 4-Me) ppm; **^{13}C-NMR** (125 MHz, $CDCl_3$, $CDCl_3$ = 77.0 ppm): δ_C = 175.7 (s, 1'-C), 168.4 (s, 1-C), 152.1 (s, carbamate-CO), 145.5 (s, 18-C), 143.2 (s, 16-C), 139.8 (s, 14-C), 132.6 (d, 12-C), 131.9 (d, 21-C), 128.6 (d, 19-C), 127.9 (d, 11-C), 127.2 (d, 17-C), 124.9 (d, 13-C), 123.2 (s, 20-C), 88.3 (d, 10-C), 80.8 (d, 9-C), 76.6 (d, 3-C), 74.2 (d, 7-C), 66.1 (d, 5-C), 60.4 (s, 4-C), 56.7 (q, 10-OMe), 46.7 (t, 15-C), 38.8 (d, 6-C), 37.5 (q, N-Me), 33.8 (t, 8-C), 33.3 (d, 2'-C), 29.7 (t, 2-C), 19.9 (q, 3'-C), 17.9 (q, 3'-C), 15.9 (q, 14-Me), 14.6 (q, 6-Me), 12.4 (q, 4-C) ppm; **HRMS (ESI)** for $C_{31}H_{41}N_2O_8BrNa$ $[M+Na]^+$: calculated 671.1944, found 671.1934.

20-Bromo-ansamitocin derivative 22c

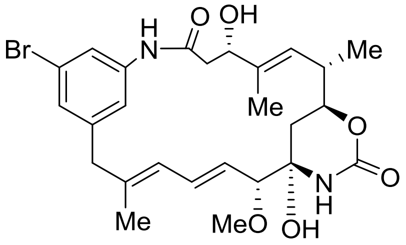

22c

NMR spectra of **22c** showed strong signal broadening which we ascribe to the missing *N*-methyl group. A higher temperature (325 K) only gave a slight improvement of resolution. Still, the ^{1}H-NMR spectrum and characteristic chemical shifts for selected protons strongly indicated the existence of the desired derivative. Therefore, these signals are listed below. ^{1}H-NMR signals were characteristic for ansamitocins and signals except 2-CH_2, 3-H, 4-CH_3, 5-H, 6-H, 8-CH_2, 11-H, 14-H, 22-H and 24-H were assigned.

^{1}H-NMR (400 MHz, $CDCl_3$, $CHCl_3$ = 7.260 ppm): δ_H = 7.36 – 7.34 (1H, s, Ar-H), 7.32 – 7.30 (1H, s, Ar-H), 7.17 – 7.15 (1H, s, Ar-H), 6.53 – 6.35 (1H, m, 12-H), 6.21 – 6.13 (1H, bs, 9-NH), 6.12 – 6.05 (1H, m, 13-H), 4.29 – 4.21 (1H, m, 7-H), 3.50 (1H, d, *J* = 9.3 Hz, 10-H), 3.43 (1H, d, *J* = 15.8 Hz, 15-H_a), 3.35 (3H, s, 10-OMe), 3.30 (1H, d, *J* = 15.8 Hz, 15-H_b), 1.19 (3H, d, *J* = 6.5 Hz, 6-Me) ppm. **HRMS (ESI)** for $C_{26}H_{33}N_2O_6BrNa$ $[M+Na]^+$: calculated 571.1420, found 571.1419.

20-Bromo-ansamitocin derivative 22e

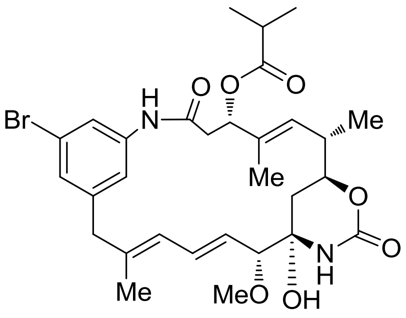

22e

NMR spectra of **22e** showed strong signal broadening which we ascribe to the missing *N*-methyl group. A higher temperature (325 K) only gave a slight improvement of resolution. ^{1}H-NMR signals were characteristic for ansamitocins and all signals except 2-CH_2, 8-CH_2, and 6-H were assigned.

^{1}H-NMR (400 MHz, $CDCl_3$, $CHCl_3$ = 7.260 ppm): δ_H = 7.40 (1H, s, amide-NH), 7.26 (1H, s, Ar-H), 7.10 (1H, s, Ar-H), 6.97 (1H, s, Ar-H), 6.52 (1H, dd, J = 15.1, 10.8 Hz, 12-H), 6.22 (1H, s, 9-NH), 6.08 – 6.00 (1H, m, 13-H), 5.57 (1H, dd, *J* = 15.1, 9.1 Hz,

11-H), 5.49 – 5.41 (1H, m, 3-H or 5-H), 5.30 – 5.24 (1H, m, 5-H or 3-H), 4.37 (1H, pt, *J* = 10.5 Hz, 7-H), 3.52 (1H, d, *J* = 9.1 Hz, 10-H), 3.42 (1H, d, *J* = 14.7 Hz, 15-H_a), 3.35 (3H, s, 10-OMe), 3.17 (1H, d, *J* = 14.7 Hz, 15-H_b), 3.05 (1H, bs, 9-OH), 2.61 (1H, sep, *J* = 7.0 Hz, 2'-H), 1.75 (3H, s, 14-Me or 4-Me), 1.66 (3H, s, 14-Me or 4-Me), 1.21 (3H, d, J = 6.9 Hz, 6-Me), 1.20 (3H, d, *J* = 7.0 Hz, 3'-H_a), 1.18 (3H, d, *J* = 7.0 Hz, 3'-H_b) ppm; not assigned: 2-CH_2, 8-CH_2 and 6-H.**HRMS (ESI)** for $C_{30}H_{39}N_2O_7BrNa$ $[M+Na]^+$: calculated 641.1838, found 641.1844.

20-Bromo-19-deschloro-ansamitocin derivatives 22b, 22d, 22f

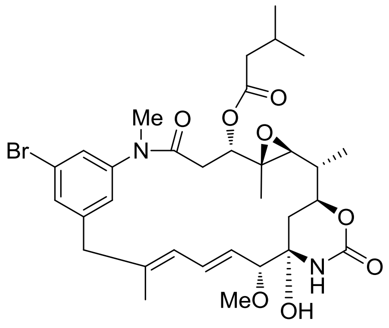

22b

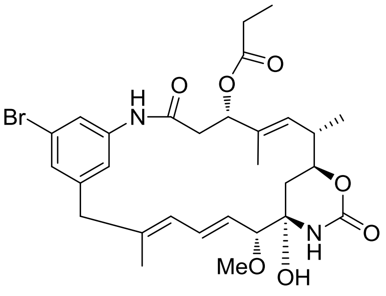

22d

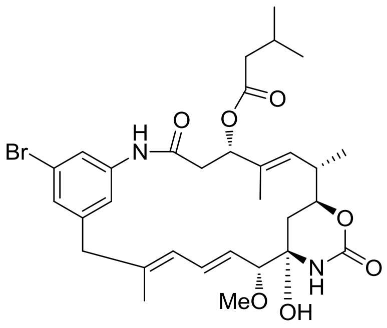

22f

22b: HRMS(ESI) for $C_{32}H_{43}N_2O_8BrNa$ $[M + Na]^+$: calculated 685.2100, found 685.2103.
22d: HRMS(ESI) for $C_{29}H_{37}N_2O_7BrNa$ $[M + Na]^+$: calculated 627.1682, found 627.1679.
22f: HRMS(ESI) for $C_{31}H_{41}N_2O_7BrNa$ $[M + Na]^+$: calculated 655.1995, found 655.1999.

Detoxification products 22g,h

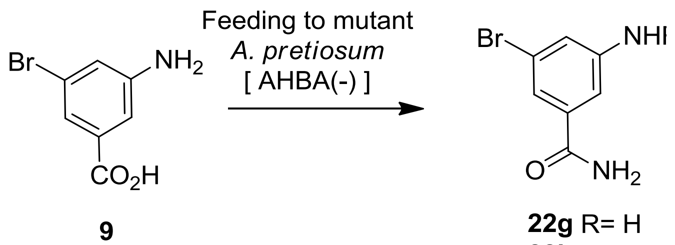

9

22g R= H
22h R= Ac

3-Amino-5-bromobenzamide 22g

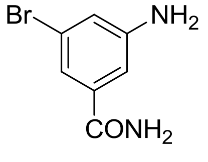

22g

^{1}H-NMR (400 MHz, $CDCl_3$, $CHCl_3$=7.260 ppm): δ_H = 7.23 (1H, pt, *J* = 1.5 Hz, Ar-H), 7.07 (1H, pt, *J* = 1.2 Hz, Ar-H), 6.96 (1H, pt, *J* = 2.1 Hz, Ar-H), 5.95 (1H, bs, $CONH_2$), 5.55 (1H, bs, $CONH_2$), 3.88 (2H, bs, NH_2) ppm; **HRMS (ESI)** for $C_7H_6N_2OBr$ $[M-H]^-$: calculated 212.9663, found 212.9668.

3-Acetamino-5-bromobenzamide 22h

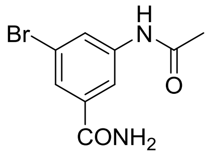

22h

^{1}H-NMR (400 MHz, MeOD, CD_2HOD=3.310 ppm): δ_H = 8.06 (1H, pt, *J* = 1.9 Hz, Ar-H), 7.92 (1H, pt, *J* = 1.5 Hz, Ar-H), 7.72 (1H, pt, *J* = 1.7 Hz, Ar-H), 2.14 (3H, s, Ac-CH_3) ppm. **HRMS (ESI)** for $C_7H_6N_2OBr$ $[M+H]^+$: calculated 256.9926, found 256.9934.

2.4 Alkynyl-substituted ansamitocin derivatives 23a–f

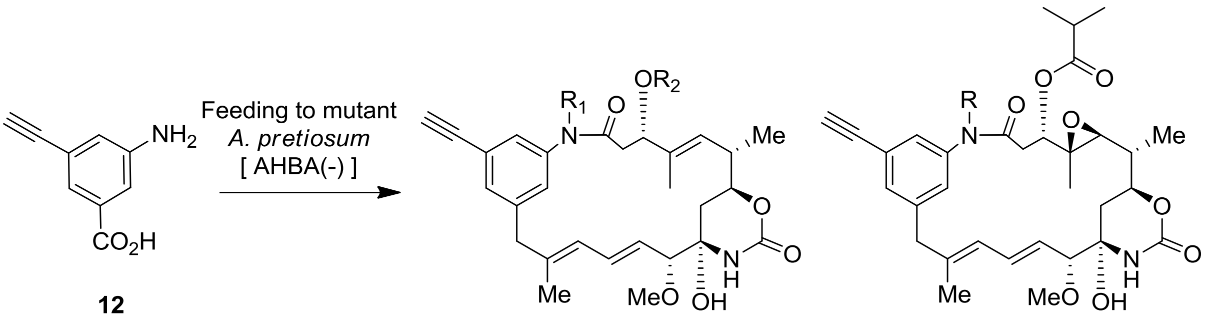

23a R^1= R^2= H
23b R^1= H, R^2= $C(O)CH_2CH_3$
23c R^1= H, R^2= $C(O)CH(CH_3)_2$
23d R^1= Me, R^2= $C(O)CH(CH_3)_2$

23e R= H
23f R= Me

Continuous complementation of a growing culture (5 L) of the AHBA blocked mutant of *A. pretiosum* with 3-amino-5-ethynylbenzoic acid (**12**) (1.2 g, 1.5 mmol/L medium) dissolved in DMSO according to the general protocol over a period of 5 days provided a crude ethyl acetate extract. This extract was prepurified by column chromatography (silica gel, ethyl acetate) and size exclusion chromatography and finally purified by HPLC (C18-SP) using a methanol-water gradient (20:80 → 100% methanol; 90 min). The fractions were further purified by HPLC (CN-SP) using an acetonitrile-water gradient (5:95 → 100% acetonitrile; 100 min) gave two ansamitocin derivatives **23f** (15 mg, 0.34%) and **23c** (2.5 mg, 0.06%). MS-analysis and retention times indicated formation of four additional ansamitocin derivatives **23a**, **23b**, **23d** and **23e**. These showed characteristic fragmentation patterns in the MS as established for the ansamitocins.

20-Ethynyl-19-deschloransamitocin P-3 (23f)

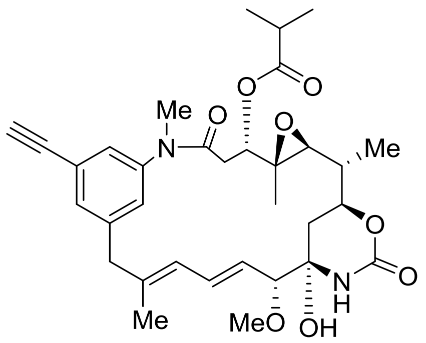

23f

^{1}H-NMR (500 MHz, MeOD, CD_2HOD = 3.310 ppm): δ_H = 7.36 (1H, bs, Ar-H), 7.26 (1H, bs, Ar-H), 7.19 (1H, bs, Ar-H), 6.48 (1H, dd, *J* = 15.4, 11.0 Hz, 12-H), 6.22 (1H, bs, NH), 6.20 (1H, d, *J* = 11.0 Hz, 13-H), 5.50 (1H, dd, *J* = 15.4, 9.0 Hz, 11-H), 4.87 (1H, dd, *J* = 12.0, 2.9 Hz, 3-H), 4.28 (1H, ddd, *J* = 12.2, 1.9, 10.4 Hz, 7-H), 3.53 (1H, d, *J* = 9.0 Hz, 10-H), 3.52 (1H, d, *J* = 13.1 Hz, 15-H_a), 3.36 (3H, s, 10-OMe), 3.27 (1H, d, *J* = 13.1 Hz, 15-H_b), 3.21 (3H, s, 1-NMe), 3.19 (1H, s, 23-H), 3.04 (1H, bs, 9-OH), 2.98 (1H, d, *J* = 9.7 Hz, 5-H), 2.69 (1H, dd, *J* = 14.0, 12.0 Hz, 2-H_a), 2.64 (1H, sep, *J* = 7.0 Hz, 2'-H), 2.23 (1H, dd, *J* = 14.0, 2.9 Hz, 2-H_b), 1.69 (3H, s, 14-Me), 1.64 (1H, d, *J* = 13.5 Hz, 8-H_a), 1.47 (1H, ddq, *J* = 10.4, 9.7, 6.3 Hz, 6-H), 1.32 (3H, d, *J* = 6.3 Hz, 6-Me), 1.29 (3H, d, *J* = 7.0 Hz, 3'-H_a), 1.25 (1H, d, *J* = 13.5, 8-H_b), 1.23 (3H, d, *J* = 7.0 Hz, 3'-H_b) 0.85 (3H, s, 4-Me) ppm; **^{13}C-NMR** (125 MHz, $CDCl_3$, $CDCl_3$=77.00 ppm): δ_C = 175.8 (s, 1'-C), 168.5 (s, 1-C), 152.1 (s, Carbamat-CO), 144.5 (s, Ar-C), 141.7 (s, Ar-C), 140.1 (s, 14-C), 132.7 (d, 12-C), 132.3 (d, Ar-C), 129.1 (d, 2x Ar-C), 127.7 (d, 11-C), 124.7 (s, Ar-C), 124.0 (d, 13-C), 88.3 (d, 10-C), 82.1 (s, 22-C), 80.8 (d, 9-C), 78.9 (d, 23-C), 76.5 (d, 3-C), 74.2 (d, 7-C), 66.1 (d, 5-C), 60.4 (s, 4-C), 56.7 (q, 10-OMe), 46.7 (t, 15-C), 38.8 (d, 6-C), 37.5 (q, N-Me), 35.7 (t, 8-C), 33.8 (d, 2'-C), 33.3 (t, 2-C), 20.0 (q, 3'-C_a), 17.9 (q, 3'-C_b), 15.8 (q, 14-Me), 14.6 (q, 6-Me), 12.4 (q, 4-C) ppm; **HRMS (ESI)** for $C_{33}H_{42}N_2O_8Na$ $[M+Na]^+$: calculated 617.2839, found 617.2834.

20-Ethynyl-19-deschloransamitocin derivative (23c)

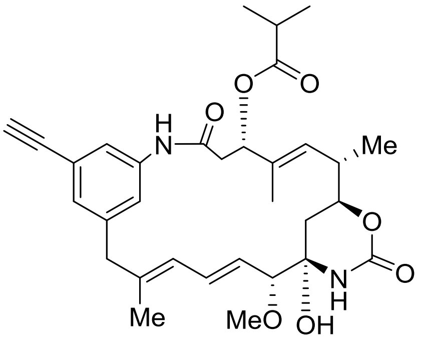

23c

NMR spectra of **23c** showed strong signal broadening due to missing *N*-methylation. ^{13}C-signals were assigned by 2D-CH correlations (HSQC). Signals of quarternary carbons could not be assigned.

^{1}H-NMR (500 MHz, MeOD, CD_2HOD = 3.310 ppm): δ_H = 8.14 (1H, bs, Ar-H), 7.66 (1H, bs, NH), 7.09 (1H, bs, Ar-H), 6.94 (1H, bs, Ar-H), 6.52 (1H, dd, *J* = 13.8, 12.0 Hz, 12-H), 6.36 (1H, bs, NH), 6.11 (1H, d, *J* = 12.0 Hz, 13-H), 5.59 (1H, dd, *J* = 13.8, 9.4 Hz, 11-H), 5.52 (1H, d, *J* = 7.6 Hz, 5-H), 5.22 (1H, d, *J* = 7.2 Hz, 3-H), 4.41 (1H, pt, *J* = 9.9 Hz, 7-H), 3.51 (1H, d, *J* = 9.1 Hz, 10-H), 3.42 (1H, d, *J* = 14.3 Hz, 15-H_a), 3.33 (3H, s, 10-OMe), 3.20 (1H, bs, 9-OH), 3.16 (1H, d, *J* = 14.3 Hz, 15-H_b), 3.06 (1H, s, 23-H), 2.85 – 2.77 (1H, m, 2-H_a), 2.69 - 2.56 (3H, m, 2-H_b, 2'-H, 6-H), 2.06 (1H, d, 8-H_a), 1.71 (3H, s, 14-Me), 1.67 (3H, s, 4-Me), 1.31 – 1.15 (10H, m, 6-Me, 2x 3'-Me, 8-H_b) ppm; **^{13}C-NMR** (125 MHz, $CDCl_3$, $CDCl_3$=77.00 ppm): δ_C = 133.1 (d, 12-C), 128.0 (d, Ar-C), 127.4 (d, 5-C), 126.9 (d, 13-C), 124.7 (11-C), 119.8 (d, 2x Ar-C), 87.1 (d, 10-C), 83.2 (d, 23-C), 77.6 (d, 7-C), 74.1 (d, 3-C), 55.8 (q, 10-OMe), 44.9 (t, 15-C), 38.8 (d, 6-C), 40.2 (t, 2-C), 37.5 (d, 6-C), 33.9 (t, 8-C), 33.8 (d, 2'-C), 18.8 (q, 2x 3'-C), 17.6 (q, 6-Me), 16.8 (q, 14-Me), 15.3 (q, 4-Me) ppm; **HRMS (ESI)** for $C_{32}H_{40}N_2O_7Na$ $[M+Na]^+$: calculated 587.2733, found 587.2736.

20-Ethynyl-19-deschloransamitocin derivatives 23a, b, d, e

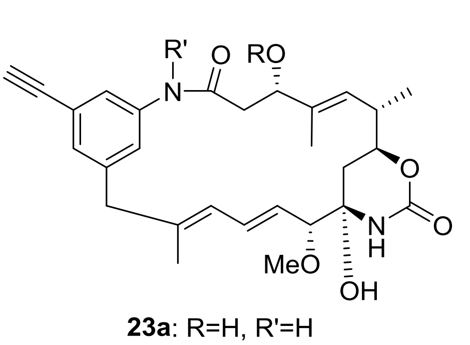

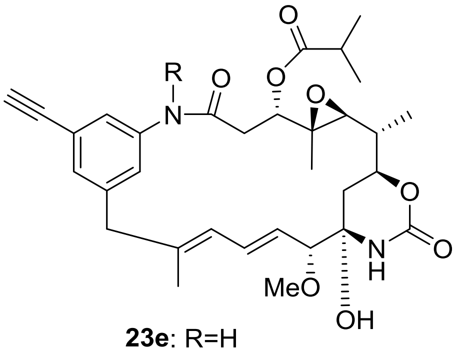

23a: R=H, R'=H

23b: R=$COCHCH_3$, R'=H

23d: R=$COCH(CH_3)_2$, R'=Me

23e: R=H

23a: HRMS(ESI) for $C_{28}H_{34}N_2O_6Na$ $[M + Na]^+$: calculated 517.2315, found 517.2315.
23b: HRMS(ESI) for $C_{31}H_{38}N_2O_7Na$ $[M + Na]^+$: calculated 573.2577, found 573.2573.
23d: HRMS(ESI) for $C_{33}H_{42}N_2O_7Na$ $[M + Na]^+$: calculated 601.2890, found 601.2875.
23e: HRMS(ESI) for $C_{32}H_{40}N_2O_8Na$ $[M + Na]^+$: calculated 603.2682, found 603.2700.

2.6 20-Propargyl-substituted ansamitocin derivatives 24a–g

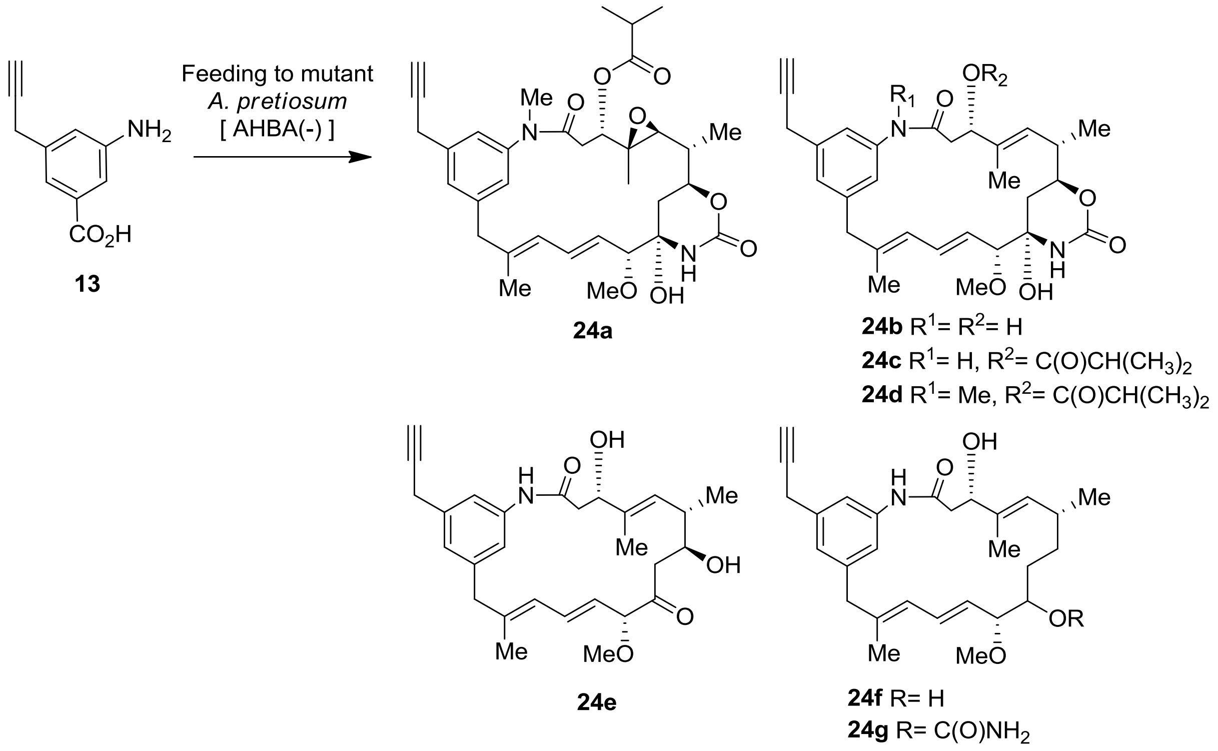

Continuous complementation of a growing culture (2 L) of the AHBA blocked mutant of *A. pretiosum* with 3-amino-5-(prop-2-yn-1-yl)benzoic acid (**13**) (1.1 g, 3 mmol/L medium) dissolved in DMSO according to the general protocol over a period of 5 days provided a crude ethyl acetate extract. This extract was prepurified by column chromatography (silica gel, ethyl acetate) and afterwards purified by HPLC (C18-SP) using a methanol-water gradient (20:80 → 100% methanol; 90 min). The fractions were further purified by HPLC (CN-SP) using an acetonitrile-water gradient (5:95 → 100% acetonitrile; 100 min). For final separation an additional HPLC (C18-SP) using an acetonitrile-water gradient (20:80 → 100% acetonitrile; 85 min) was employed. This protocol provided five ansamitocin derivatives **24b**–**d** and **24f**–**g** in 0.1% yield, which were characterized by NMR-spectroscopy. MS-analysis and retention times indicated formation of at least two additional ansamitocin derivatives **24a** and **24e**. These showed characteristic fragmentation patterns in the MS as established for the ansamitocins.

20-Propargyl-substituted ansamitocin derivative 24b

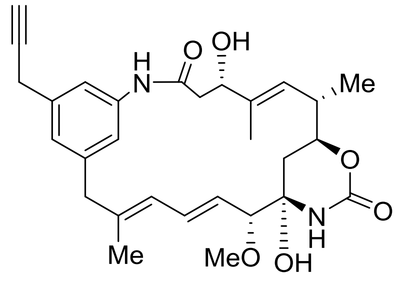

24b

Compound **24b** was isolated in too small amounts for recording ^{13}C NMR spectra and HSQC correlations.
^{1}H-NMR (500 MHz, CD_2H_3OD, CD_2HOD = 3.31 ppm): δ_H = 7.93 (1H, s, Ar-H), 6.64 (1H, s, Ar-H), 6.90 (1H, s, Ar-H), 6.64 (1H, dd, J = 15.3, 11.2 Hz, 12-H), 6.11 (1H, d, *J* = 11.2 Hz, 13-H), 5.58 (1H, dd, *J* = 15.3, 9.4 Hz, 11-H), 5.46 (1H, d, *J* = 8.4 Hz, 5-H), 4.30 (1H, pt, *J* = 10.8 Hz, 7-H), 4.21 (1H, d, *J* = 6.1 Hz, 3-H), 3.57 (1H, d, *J* = 9.4 Hz, 10-H), 3.55 (2H, d, *J* = 2.8 Hz, 22-CH_2), 3.42 (1H, bs, 15-H_a), 3.34 (3H, s, 10-OMe), 3.20 (1H, bs, 15-H_b), 2.84 (1H, dd, *J* = 15.5, 2.5 Hz, 2-H_a), 2.72 (1H, dd, *J* = 15.5, 7.0 Hz, 2-H_b), 2.68 – 2.62 (1H, m, 6-H), 2.51 (1H, t, *J* = 2.8 Hz, 24-H), 1.97 (1H, d, *J* = 13.3 Hz, 8-H_a), 1.68 (3H, s, 14-CH_3), 1.66 (3H, s, 4-CH_3), 1.45 (1H, d, *J* = 13.3 Hz, 8-H_b), 1.14 (3H, d, *J* = 6.5 Hz, 6-Me) ppm; **HRMS (ESI)** for $C_{29}H_{37}N_2O_6$ $[M+H]^+$: calculated 509.2652, found 509.2665.

20-Propargyl-19-deschloro-des-*N*-methyl-desepoxyansamitocin (24c)

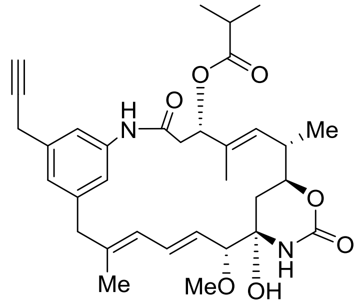

24c

Compound **24c** was isolated in too small amounts for detecting ^{13}C signals and HSQC correlations. Nevertheless, the spectrum indicated the existence of the desired derivative due to significant shifts for protons in ^{1}H spectrum. Therefore these signals are listed below.

^{1}H-NMR (500 MHz, CD_2HOD, $CD_{2H}OD$ = 3.31 ppm): δ_H = 7.97 (1H, s, Ar-H), 6.92 (1H, s, Ar-H), 6.87 (1H, s, Ar-H), 6.68 (1H, dd, *J* = 15.2, 11.6 Hz, 12-H), 6.10 (1H, d, *J* = 11.6 Hz, 13-H), 5.59 (1H, dd, *J* = 15.2, 8.3 Hz, 11-H), 5.37 – 5.27 (2H, m, 5-H, 3-H), 4.27 (1H, pt, *J* = 10.7 Hz, 7-H), 3.58 (2H, bs, 22-CH_2), 3.37 (3H, s, 10-OMe), 1.76

(3H, s, 14-Me), 1.73 (3H, s, 4-Me), 1.19 (3H, d, J = 7.0 Hz, 3'-H_a), 1.18 (3H, d, J = 7.0 Hz, 3'-H_b), 1.11 (3H, d, J = 6.2 Hz, 6-Me) ppm; not assigned: 10-H, 15-CH_2, 24-H, 6-H, 2'-H, 2-CH_2 and 8-CH_2. **HRMS (ESI)** for $C_{33}H_{42}N_2O_7Na$ $[M+Na]^+$: calculated 601.2890, found 601.2878.

20-Propargyl-19-deschloro-desepoxyansamitocin P-3 (24d)

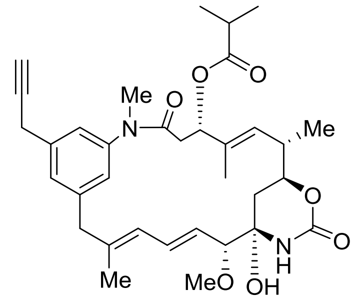

24d

Compound **24d** was isolated in too small amounts for detecting ^{13}C signals and HSQC correlations. Therefore the below listed signals were assigned via comparison with the typical ^{1}H-shifts and COSY correlations.

^{1}H-NMR (500 MHz, CD_2HOD, CD_2HOD=3.31 ppm): δ_H = 7.31 (1H, s, Ar-H), 7.23 (1H, s, Ar-H), 7.10 (1H, s, Ar-H), 6.56 (1H, dd, J = 15.2, 10.7 Hz, 12-H), 5.97 – 5.87 (1H, m, 13-H), 5.42 – 5.34 (2H, m, 11-H, 3-H), 5.15 – 5.11 (1H, m, 5-H), 4.02 (1H, pt, J = 10.0 Hz, 7-H), 3.69 (2H, d, J = 2.5 Hz, 22-H), 3.55 (1H, d, J = 8.9 Hz, 10-H), 3.48 (1H, d, J = 13.5 Hz, 15-H_a), 3.34 (3H, s, 10-OMe), 3.31 (1H, 15-H_b under CD_2HOD-signal), 3.22 (3H, s, N-Me), 2.63 – 2.56 (2H, m, 24-H, 2'-H), 2.54 – 2.47 (2H, m, 6-H, 2-H_a), 2.38 – 2.31 (1H, m, 2-H_b), 1.84 (6H, bs, 4-CH_3, 14-CH_3), 1.51 (1H, dd, J = 14.3, 2.4 Hz, 8-H_a), 1.33 – 1.31 (1H, m, 8-H_b), 1.19 (3H, d, J = 7.1 Hz, 3'-H_a), 1.17 (3H, d, J = 7.1 Hz, 3'-H_b), 1.07 (3H, d, J = 6.4 Hz, 6-Me) ppm; **HRMS (ESI)** for $C_{34}H_{44}N_2O_7Na$ $[M+Na]^+$: calculated 615.3046, found 615.3038.

20-Propargyl-substituted proansamitocin derivate 24f

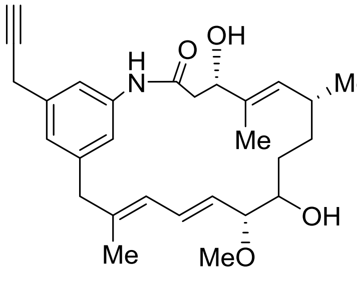

24f

^{13}C-signals were assigned via HSQC and HMBC correlations. Signals for quarternary aromatic carbons and carbonyl of amide could not be assigned.

^{1}H-NMR (500 MHz, CD_2HOD, CD_2HOD= 3.310 ppm): δ_H = 7.57 (1H, s, Ar-H), 6.98 (1H, s, Ar-H), 6.94 (1H, s, Ar-H), 6.52 (1H, dd, *J* = 15.2, 11.4 Hz, 12-H), 6.01 (1H, d, *J* = 11.4 Hz, 13-H), 5.40 (1H, dd, *J* = 15.2, 7.5 Hz, 11-H), 5.37 (1H, d, *J* = 7.5 Hz, 5-H), 4.33 (1H, pt, *J* = 5.7 Hz, 3-H), 3.56 (2H, bs, 22-H), 3.45 – 3.42 (2H, m, 10-H, 9-H), 3.39 (1H, bs, 15-H_a), 3.36 (1H, bs, 15-H_b), 3.26 (3H, s, 10-OMe), 2.70 – 2.67 (2H, m, 2-CH_2), 2.51 (1H, bs, 24-H), 2.36 – 2.26 (1H, m, 6-H), 1.72 (3H, s, 14-CH_3), 1.65 (3H, s, 4-CH_3), 1.54 – 1.48 (2H, m, 7-CH_2), 1.36 – 1.29 (2H, m, 8-CH_2), 0.83 (3H, d, *J* = 6.6 Hz, 6-Me) ppm; **^{13}C-NMR** (125 MHz, CD_2HOD, CD_2HOD = 49.00 ppm): δ_C = 138.6 (s, 14-C), 134.0 (s, 4-C), 131.5 (d, 5-C), 130.6 (d, 12-C), 127.9 (d, 11-C), 126.2 (d, 13-C), 124.2 (d, Ar-C), 118.8 (d, 17-C), 116.9 (d, Ar-C), 87.1 (d, 10-C), 74.5 (d, 9-C), 80.8 (s, 23-C), 72.8 (d, 3-C), 69.9 (d, 24-C), 55.2 (q, 10-OMe), 45.1 (t, 15-C), 40.8 (t, 2-C), 31.8 (d, 6-C), 30.0 (t, 7-C), 29.4 (t, 8-C), 23.2 (t, 22-C), 19.7 (q, 6-CH3), 15.6 (q, 14-CH_3), 11.7 (q, 4-CH_3) ppm; **HRMS (ESI)** for $C_{28}H_{37}NO_4Na$ $[M+Na]^+$: calculated 474.2620, found 474.2643.

20-Propargyl-substituted proansamitocin derivate 24g

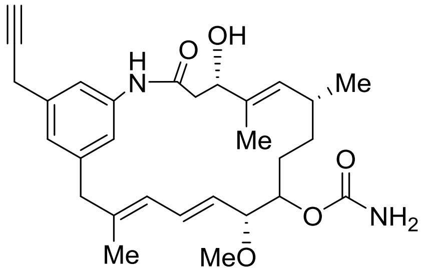

24g

^{13}C-signals were assigned via HSQC and HMBC correlations. Signals for quarternary aromatic carbons and carbonyl of carbamate and amide could not be assigned.

^{1}H-NMR (500 MHz, CD_2HOD, CD_2HOD = 3.31 ppm): δ_H = 7.54 (1H, s, Ar-H), 6.98 (1H, s, Ar-H), 6.95 (1H, s, Ar-H), 6.52 (1H, dd, *J* = 15.0, 11.0 Hz, 12-H), 6.02 (1H, d, *J* = 11.0 Hz, 13-H), 5.47 (1H, dd, *J* = 15.0, 7.5 Hz, 11-H), 5.33 (1H, d, *J* = 8.0 Hz, 5-H), 4.72 – 4.67 (1H, m, 9-H), 4.36 (1H, dd, *J* = 7.9, 3.7 Hz, 3-H), 3.69 (1H, pt, *J* = 7.2 Hz, 10-H), 3.56 (2H, bd, *J* = 1.8 Hz, 22-H), 3.33 (1H, 15-H_a, under CD_2HOD-signal), 3.31 (1H, 15-H_b, under CD_2HOD-signal), 3.29 (3H, s, 10-OMe), 2.70 – 2.62 (2H, m, 2-CH_2), 2.51 (1H, bs, 24-H), 2.32 – 2.23 (1H, m, 6-H), 1.71 (3H, s, 14-CH_3), 1.65 (3H, s, 4-CH_3), 1.60 – 1.55 (1H, m, 8-H_a), 1.45 – 1.41 (1H, m, 8-H_b), 1.39 – 1.32 (2H, m, 7-CH_2), 0.79 (3H, d, *J* = 6.7 Hz, 6-Me) ppm; **^{13}C-NMR** (125 MHz, CD_2HOD, CD_2HOD = 49.0 ppm): δ_C = 138.6 (s, 14-C), 134.5 (s, 4-C), 132.2 (d, 5-C), 130.1 (d, 12-C), 127.6 (d, 11-C), 126.2 (d, 13-C), 124.14 (d, Ar-C), 118.8 (d, 17-C), 117.7 (d, Ar-C), 83.7 (d, 10-C), 81.3 (s, 23-C), 74.9 (d, 9-H), 71.9 (d, 3-C), 70.4 (d, 24-C), 55.4 (q, 10-OMe), 44.5 (t, 15-C), 41.1 (t, 2-C), 32.4 (t, 8-C), 31.3 (d, 6-C), 31.5 (t, 7-C), 23.0 (t, 22-C),

19.3 (q, 6-CH_3), 15.6 (q, 14-CH_3), 11.4 (q, 4-CH_3) ppm; **HRMS (ESI)** for $C_{29}H_{38}N_2O_5Na$ $[M+Na]^+$: calculated 517.2678, found 509.2688.

20-Propargyl-19-deschloroansamtitocin derivatives 24a and 24e

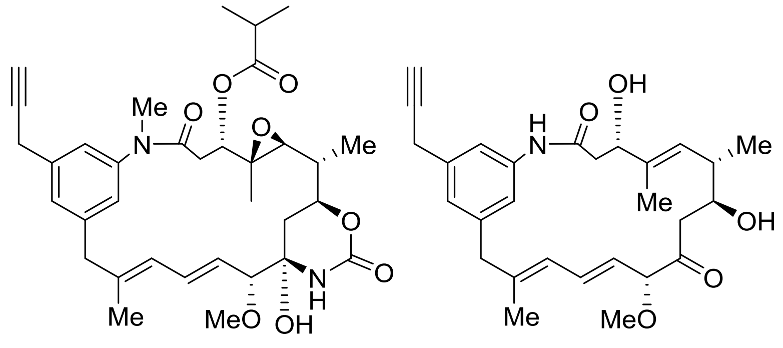

24a **24e**

24a: HRMS (ESI) for $C_{34}H_{44}N_2O_7Na$ $[M + Na]^+$: calculated 615.3046, found 615.3038.
24e: HRMS (ESI) for $C_{28}H_{35}NO_5Na$ $[M + Na]^+$: calculated 488.2413, found 488.2429.

2.7 20-Vinylansamitocin derivatives 25

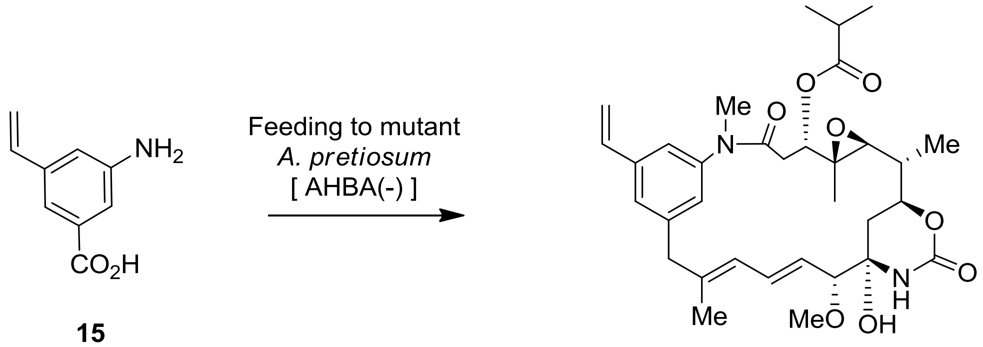

15 **25**

Continuous (over 5 days) complementation of a growing culture (50 mL) of the AHBA blocked mutant of *A. pretiosum* with 3-amino-5-vinylbenzoic acid (**15**) (2*12 mg, 2*75 µmol) dissolved in DMSO according to the general protocol provided a crude ethyl acetate extract. This extract was prepurified by column chromatography (silica gel; ethyl acetate) and analyzed by UPLC-MS. MS-analysis indicated the formation of new ansamitocin derivative **25** as judged by characteristic fragmentation patterns that have been established for the ansamitocins.

25: HRMS (ESI) for $C_{33}H_{44}N_2O_8Na$ $[M + Na]^+$: calculated 619.2995 found 619.2994.

2.8 Linker 26a-b

3-Azidopropanol

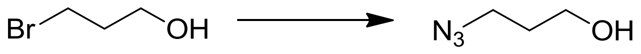

3-Bromopropanol (45 µL, 0.5 mmol, 1 equiv) was dissolved in DMF (1 mL) and sodium azide (39 mg, 0.6 mmol, 1.2 equiv) was added. The reaction mixture was stirred for 20 h at room temperature before it was hydrolysed by addition of H_2O. The crude product was extracted with Et_2O, dried with $MgSO_4$ and concentrated in vacuo. The product (110 mg) was isolated as a colorless oil in 89% yield (contaminated with byproducts; determined by NMR).

^{1}H-NMR ($CDCl_3$, 400 MHz, $CHCl_3$= 7.26 ppm): δ_H = 3.74 (2H, t, *J* = 6.3 Hz, CH_2OH), 3.44 (2H, t, *J* = 6.3 Hz, CH_2N_3), 1.93 (1H, bs, OH), 1.82 (2H, p, *J* = 6.3 Hz, 2-CH_2) ppm; **^{13}C-NMR** ($CDCl_3$, 100 MHz, $CDCl_3$ = 77.0 ppm): 59.8 (t, CH_2OH), 48.5 (t, CH_2N_3), 31.5 (t, 2-CH_2) ppm.

3-Azidopropanthiol 26a

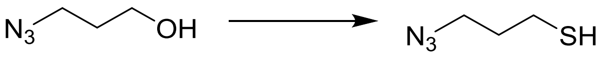

26a

3-Azidopropanol (28 mg, 0.28 mmol, 1 equiv) was dissolved in THF and trifluoroacetic acid anhydride (43 µL, 0.31 mmol, 1.1 equiv) was added. The mixture was stirred for 30 min at room temperature before removing access of the anhydride in vacuo. The resulting ester was redissolved in THF and LiBr (26.9 mg, 0.31 mmol, 1.1 equiv) as well as HMPA (234 µL, 1.3 mmol, 4.8 equiv) were added. The reaction mixture was stirred 5 h at 70 °C under an argon atmosphere before it was hydrolysed by addition of H_2O. The product was extracted with Et_2O, dried with $MgSO_4$ and concentrated in vacuo. The resulting azido bromide, a colorless oil, which was still contaminated with DMF and HMPA (determined by NMR), was directly used for the next step.

Crude product obtained from the bromination step (101 mg) was dissolved in ethanol (1 mL) and thiourea (23.6 mg, 0.31 mmol, 1.1 equiv) dissolved in ethanol (1 mL) was added. The reaction mixture was stirred for 20 h at 80 °C before it was hydrolysed by addition of $NaOH_{aq}$ (5%). The mixture was acidified by addition of aqueous H_2SO_4 (10%) and stirring was continued for 20 min at room temperature. The product was extracted with CH_2Cl_2, the organic phase was dried with $MgSO_4$ and concentrated in vacuo. The crude product was purified by column chromatography (silica gel, petroleum ether/ethyl acetate = 10:1). 3-Azidopropanthiol **26a** (20 mg, 0.17 mmol, 61%) was received as a colorless oil with a characteristic smell of rubber tires.

^{1}H-NMR ($CDCl_3$, 400 MHz, $CHCl_3$= 7.26 ppm): δ_H = 3.43 (2H, t, *J* = 6.5 Hz, CH_2N_3), 2.75 (2H, t, *J* = 7.2 Hz, CH_2SH), 1.99 (2H, p, *J* = 6.7 Hz, 2-CH_2) ppm; **^{13}C-NMR**

($CDCl_3$, 100 MHz, $CDCl_3$ = 77.0 ppm): δ_C = 49.7 (t, CH_2N_3), 35.2 (2-CH_2), 28.3 (t, 2-CH_2SH) ppm.

3-Azido-2,2-dimethylpropan-1-ol

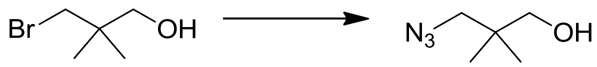

3-Azido-2,2-dimethylpropan-1-ol was prepared according to the procedure described for 3-azidopropanol. The product (137 mg) was isolated as a colorless oil contaminated with (2.6:1) in 86% yield (determined by NMR).
^{1}H-NMR ($CDCl_3$, 400 MHz, $CHCl_3$= 7.26 ppm): δ_H = **^{1}H-NMR** ($CDCl_3$, 400 MHz, $CHCl_3$= 7.26 ppm): d_H = 3.45 (2H, s, CH_2OH), 3.38 (2H, s, CH_2N_3), 1.82 (1H, bs, OH), 1.01 (6H, s, 2-CH_3) ppm; **^{13}C-NMR** ($CDCl_3$, 100 MHz, $CDCl_3$ = 77.0 ppm): δ_C = 69.1 (t, CH_2OH), 43.1 (t, CH_2N_3), 22.9 (q, 2-CH_3) ppm.

3-Azido-2,2-dimethylpropane-1-thiol

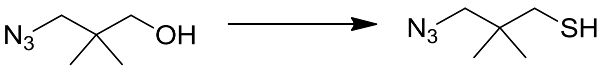

3-Azido-2,2-dimethylpropane-1-thiol was prepared according to the procedure described for 3-azidopropanthiol. 3-Azido-2,2-dimethylpropane-1-thiol (31.5 mg, 0.22 mmol, 68%) was obtained as a colorless oil with a characteristic smell of rubber tires.
^{1}H-NMR ($CDCl_3$, 400 MHz, $CHCl_3$= 7.26 ppm): δ_H = 3.48 (2H, s, CH_2SH), 3.39 (2H, s, CH_2N_3), 1.03 (6H, s, 2x 2-CH_3) ppm; **^{13}C-NMR** ($CDCl_3$, 100 MHz, $CDCl_3$ = 77.0 ppm): δ_C = 69.2 (t, CH_2SH), 43.0 (t, CH_2N_3), 36.7 (s, 2-C), 22.9 (q, 2x2-CH_3) ppm.

***S*-(3-Azido-2,2-dimethylpropyl) ethanethioate 26b**

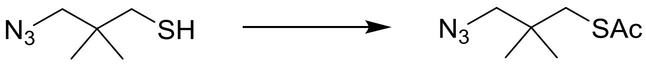

26b

3-Azido-2,2-dimethylpropane-1-thiol (29 mg, 0.2 mmol, 1 equiv) was dissolved in THF (0.4 mL), then acetyl chloride (29 µL, 0.4 mmol, 2 equiv) and triethylamine (57 µL, 0.4 mmol, 2 equiv) were added and the mixture was stirred for 20 h at room temperature. Hydrolysis was achieved by addition of H_2O which was followed by extraction with Et_2O. The organic phase was dried with $MgSO_4$ and concentrated in vacuo. *S*-(3-Azido-2,2-dimethylpropyl) ethanethioate **26b** (23 mg, 0.12 mmol, 61%) was isolated in form of a colorless volatile liquid.

^{1}H-NMR ($CDCl_3$, 400 MHz, $CHCl_3$= 7.26 ppm): δ_H = 3.93 (2H, s, CH_2SAc), 3.34 (2H, s, CH_2N_3), 2.07 (3H, s, Ac-CH_3), 1.05 (6H, 2x2-CH_3) ppm; **^{13}C-NMR** ($CDCl_3$, 100

MHz, $CDCl_3$ = 77.0 ppm): δ_C = 69.8 (t, CH_2SAc), 42.3 (t, CH_2N_3), 35.3 (s, 2-C), 23.2 (q, 2x2-CH_3), 20.8 (q, Ac-CH_3) ppm.

Ansamitocin derivate 27b

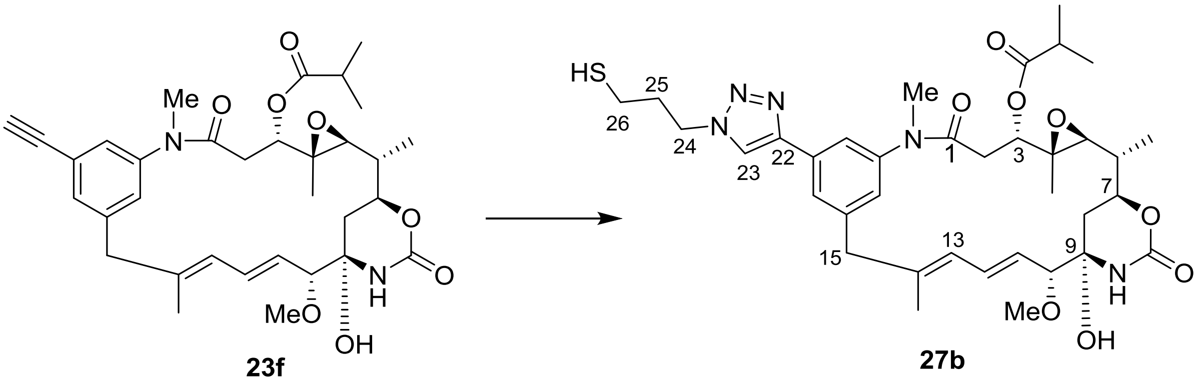

20-Propargyl-19-deschloransamitocin P3 **23f** (1mg, 1.7 µmol; 1 equiv) was dissolved in a mixture of *t*-BuOH/THF/H_2O (1:2:3, 0.5 mL). Then, 4-azidopropanthiol **26a** (0.3 mg, 2.5 µmol; 1.5 equiv) and CuBr·SMe_2 (0.1 mg, 0.5 µmol; 0.25 equiv) were added and the mixture was stirred 40 h at room temperature before it was terminated by addition of phosphate buffer (pH 7). The crude product was extracted with ethyl acetate and after concentration in vacuo it was directly purified by HPLC (C18-SP) using an acetonitrile-water gradient (20:80 → 100% acetonitrile; 85 min). The disulfide of **27b** (0.7 mg) was obtained. It was dissolved in methanol and dithiothreitol was added under an argon atmosphere. After stirring for 4 h at room temperature disulfide cleavage was accomplished as judged by LC–MS. The reaction mixture was directly subjected to HPLC purification (**C18-SP**) applying an acetonitrile-water gradient (20:80 → 100% acetonitrile; 85 min). Compound **27b** (0.6 mg, 0.9 µmol, 53%) was isolated. Additionally, starting material **23f** (0.3 mg, 0.5 µmol, 32%) was reisolated.

Compound **27b** was isolated such small amount that hampered recording of ^{13}C spectra as well as HSQC correlations. Therefore, the signals listed below were assigned by comparison with the typical ^1H-shifts and COSY correlations assigned for compound **27c**. The product redimerized in the NMR solvent, which was detected by time dependent LC–MS measurements. Therefore, the spectra revealed two sets of signals.

^{1}H-NMR (500 MHz, MeOD, CD_2HOD = 3.31 ppm): δ_H = 8.48 (1H, d, *J* = 2.4 Hz), 7.84 (1H, bs, Ar-H), 7.68 (1H, bs, Ar-H), 7.25 (1H, bs, Ar-H), 6.65 (1H, dd, *J* = 15.0, 11.0 Hz, 12-H), 6.30 (1H, d, *J* = 11.0 Hz, 13-H), 5.55 (1H, dd, *J* = 15.0, 9.0 Hz, 11-H), 4.76 – 4.73 (1H, m, 3-H), 4.58 (2H, t, *J* = 6.8 Hz, 24-H), 4.22 (1H, pt, *J* = 10.9 Hz, 7-H), 3.65 – 3.57 (2H, m, 10-H, 15-H_a), 3.37 (3H, s, 10-OMe), 3.31 (1H, m, under CD_2HOD-signal, 15-H_b), 3.26 (3H, s, 1-NMe), 2.87 – 2.81 (2H, m, 5-H, 2-H_a), 2.79 – 2.72 (3H, m, 25-CH_2, 2'-H), 2.37 (2H, p, *J* = 6.8 Hz, 26-H), 2.26 – 2.20 (1H, m, 2-H_b), 1.74 (3H, s, 14-Me), 1.63 – 1.50 (1H, m, 6-H), 1.25 – 1.20 (9H, m, 6-Me, 2x 3'-Me),

0.90 (3H, s, 4-Me) ppm; not assigned: signals of 8-CH_2; **HRMS (ESI)** for $C_{36}H_{50}N_5O_8S$ $[M+H]^+$: calculated 712.3380, found 712.3385.

Ansamitocin derivate 27c

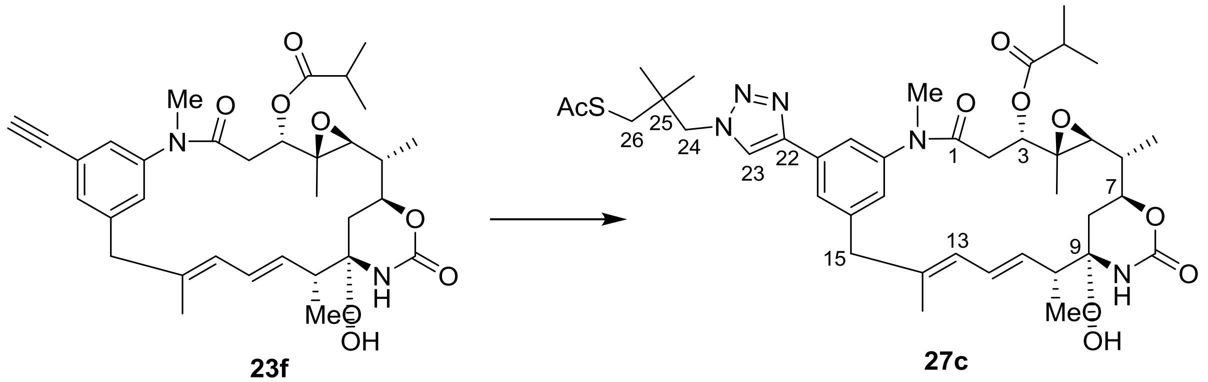

23f **27c**

Compound **27c** was prepared according to the procedure described for **27b**. Compound **27c** (0.8 mg, 1 µmol, 59%) was isolated. Additionally, starting material **23f** (0.2 mg, 0.3 µmol, 20%) was reisolated.

^{1}H-NMR (500 MHz, MeOD, CD_2HOD = 3.31 ppm): δ_H = 7.72 (1H, dd, *J* = 7.6, 1.7 Hz, triazol-H), 7.56 (1H, bs, Ar-H), 7.47 (1H, bs, Ar-H), 7.34 (1H, bs, Ar-H), 6.65 (1H, dd, *J* = 15.2, 11.0 Hz, 12-H), 6.29 (1H, d, *J* = 11.0 Hz, 13-H), 5.55 (1H, dd, *J* = 15.2, 9.0 Hz, 11-H), 4.74 (1H, dd, *J* = 12.0, 2.9 Hz, 3-H), 4.22 (1H, ddd, *J* = 11.1, 10.8, 2.4 Hz, 7-H), 3.89 (2H, s, 26-H), 3.59 (1H, d, *J* = 13.3 Hz, 15-H_a), 3.58 (1H, d, *J* = 9.0 Hz, 10-H), 3.36 (3H, s, 10-OMe), 3.36 - 3.34 (1H, m, 15-H_b), 3.22 (3H, s, 1-NMe), 2.87 (2H, s, 24-H), 2.85 (1H, d, *J* = 9.7 Hz, 5-H), 2.77 – 2.70 (2H, m, 2-H_a, 2'-H), 2.23 (1H, dd, *J* = 14.1, 2.5 Hz, 2-H_b), 1.72 (3H, s, 14-Me), 1.64 – 1.50 (2H, m, 8-H_a, 6-H), 1.27 (3H, d, *J* = 7.2 Hz, 3'-H_a), 1.24 (3H, d, *J* = 6.4 Hz, 6-Me), 1.21 (3H, d, *J* = 6.6 Hz, 3'-H_b), 1.19 – 1.15 (1H, m, 8-H_b), 1.02 (6 H, s, 2x 25-CH_3), 0.93 (3H, s, 4-Me) ppm; **^{13}C-NMR** (125 MHz, MeOD, CD_2HOD=49.00 ppm): δ_C = 176.4 (s, 1'-CO), 171.3 (s, Ac-CO), 169.7 (s, amide-CO), 144.9 (s, Ar-C), 135.8 (d, 23-C), 134.3 (d, Ar-C), 133.8 (d, 12-C), 130.1 (d, 11-C), 130.0 (d, Ar-C), 129.6 (s, Ar-C), 128.4 (d, Ar-C), 126.6 (d, 13-C), 89.7 (d, 10-C), 80.4 (s, 22-C), 77.7 (d, 3-C), 75.7 (d, 7-C), 71.8 (t, 26-C), 67.5 (d, 5-C), 57.0 (q, 10-OMe), 51.4 (t, 24-C), 46.9 (t, 15-C), 39.5 (d, 6-C), 38.0 (t, 8-C), 37.8 (q, N-Me), 35.5 (s, 25-C), 35.3 (d, 2'-C), 34.2 (t, 2-C), 24.1 (q, 25-C), 20.5 (q, 3'-C), 20.3 (q, Ac-Me), 18.1 (q, 3'-C), 15.9 (q, 14-Me), 15.3 (q, 6-Me), 12.5 (q, 4-C) ppm; unassigned: carbamate-CO, 14-C, 4-C and one aromatic quarternary C-signal.

3. Cell proliferation assay

Cell lines were obtained from DMSZ (U-937 ACC 5; A-431 ACC 91) or ATCC (SK-OV-3 HTB-77; PC-3 CRL-1435). Growth inhibition was measured in microtiter plates. 60 µL of serial dilutions of the test compounds were added to 120 µL aliquots of a cell suspension (50.000/mL) in 96-well plates and incubated at 37 °C and 10% CO_2 for 5 days. MTT [3(4,5-dimethylthiazol-2-yl)2,5-diphenyltetrazolium bromide] was used to

measure growth and viability of cells which are capable of reducing it to a violet formazan product. 20 µL MTT in phosphate buffered saline (PBS) were added to a final concentration of 0.5 mg/mL. After 2 h the precipitate of formazan crystals was centrifuged, and the supernatant discarded. The precipitate was washed with PBS (100 µL) and dissolved in isopropanol (100 µL) containing 0.4% hydrochloric acid. The microplates were measured at 595 nm using an ELISA plate reader. All experiments were carried out in two parallel experiments. The percentage of viable cells was calculated as the mean with respect to the controls set to 100%. In the case of U-937 the WST-1 assay from Roche was employed.

4. References

[S1] Gottlieb, H. E.; Kotlyar, V.; Nudelman, A. *J. Org. Chem.* **1997**, *62*, 7512-7515.

[S2] Leonard, J.; Lygo, B.; Procter, G. *Praxis in der organischen Chemie*, VCH, Weinheim, **1996**

[S3] Eichner, S.; Floss, H. G., Sasse, F., Kirschning, A., *ChemBioChem* **2009**, *10*, 1801.

[S4] Knobloch, T., Harmrolfs, K., Knobloch, T., Taft, F.; Thomaszewski, B., Sasse, F., Kirschning, A. *ChemBioChem* **2011**, *12*, 540-547.

[S5] Eichner, S., Eichner, T., Floss, H. G., Fohrer, J., Hofer, E., Sasse, F., Zeilinger, C., Kirschning, A., *J. Am. Chem. Soc.*, **2012**, *134*, 1673-1679.

[S6] Harding, K. E.; May, L. M.; Dick, K. F. *J. Org. Chem*., **1975**, *40*, 1664-1665.

[S7] Yu, T.-W.; Bai, L.; Clade, D.; Hoffmann, D.; Toelzer, S.; Trinh, K. Q.; Xu, J.; Moss, S. J.; Leistner, E.; Floss, H. G., *Proc. Nat. Acad. Sci. USA* **2002**, *99*, 7968-7973.
